## [Reviewer comments · BMJ Open]

ARTICLE DETAILS

TITLE (PROVISIONAL)	The Impact on Staff of Providing Non-Invasive Advanced Respiratory Support During the Covid-19 Pandemic– A Qualitative Study in an Acute Hospital
AUTHORS	Wenzel, David; Bleazard, Lucy; Wilson, Eleanor; Faull, Christina

VERSION 1 – REVIEW

REVIEWER	Pearmain, Laurence Wellcome Trust Centre for Cell Matrix Research
REVIEW RETURNED	21-Jan-2022

GENERAL COMMENTS	This is an important area with widespread relevance to the healthcare workforce within the UK and internationally. It is sorely in need of research and I commend the authors for taking on a challenging and important topic. Overall the paper is well written, engaging and emotive without losing objectivity. The results are clear and discussion and conclusion appropriate. The study design is appropriate and outcomes clear. The results meet these outcomes. I feel the methods need to either be more detailed/explicit or thoroughly referenced. I address this in more detail below.... The following are specific issues I feel may strengthen the paper, if addressed: It may help to highlight the extent of the issue that both governments (Welsh government have issued a report in moral injury in healthcare workers) and professional bodies (BMA have issued report into moral injury) are concerned about moral injury to healthcare workers from COVID care. In the introduction it would be helpful if the authors specified that the high mortality associated with CPAP is in patients not deemed likely to benefit from invasive mechanical ventilation. In those for escalation it's role is limited to intubation avoidance. As such very few of the (generally) younger, fitter COVID-19 pneumonitis population die on CPAP, though I acknowledge managing them can be traumatic for staff in other ways. Whilst the study design appears appropriate I would recommend that the authors state why they felt a constructivist approach was the right one for this project apposed to other forms of thematic analysis.
--

	The methods are generally clear and well described, however it is unclear why, or how the group interview differed from the individual interviews. This is important, as participants may influence each other and therefore accentuate themes etc. Within the description of participants (including supplementary table) I think it is important to be more specific about the breakdown of nurse colleagues, who will have very different roles and duties- this is important to confirm diverse sampling. Within the methods "Data collection was completed when thematic saturation was achieved". Can the authors please confirm whether this was a pre-defined recruitment end-point, and how this was determined? If by a commonly accepted method then a reference could be used. Whilst some reflexivity is provided within the limitations in the discussion I feel this would be better stated more explicitly- perhaps as supplementary information alongside the specific roles they had. E.g who exactly conducted the interviews? Were both DW and LB present for all, did DW conduct some and LB some? Information about CF from a reflexive perspective may be appropriate, if they helped with interpretation/contextualizing the emerging themes for discussion. In the discussion I think it would be useful to highlight that there is no evidence/literature (that I could find/am aware) of NIV helping symptom control in COVID19 at all. It is then more obvious/reasonable to discuss NIV palliative role in COPD guidelines if the authors feel relevant (I'm not sure it can be extrapolated to COVID, but that is a matter of opinion not fact!). Having worked in the frontline I found the interviews emotive and brought me back to many of my own experiences. That is testament to how powerful this piece of work can be. It may be wise, if the editor decides to publish, to put a "trigger warning" statement early in the article. The quotations are likely to be the area of the article some readers may find difficult, so there is plenty of time to do this prior to then. Thank you for sharing this work with me, I hope the comments are helpful to the authors.
--	--

REVIEWER	Whittle, Jessica S. University of Tennessee
REVIEW RETURNED	14-Feb-2022

GENERAL COMMENTS	General / Methods: 1 - I recommend further description of demographics of participants. How do they compare to staff in general? Why 15/21 female? – make appendix A into a table 2 - Describe survey in more detail. Describe the interview process 3 - Improve/ standardize abbreviations: MDT? NARS is more typically NIPPV
--

	4 - Was any validated PTSD screening survey used? Moral injury is often somewhat quantified using these and they seem appropriate here. 5 – How severe was the impact to the participants? It was briefly noted that some required formal therapy, but was any attempt made to evaluate the extent of the injury? 6 - Further discuss the obvious significant limitations of small sample size, recall bias, etc Specific notes: Page 8 line 18 “A minority of participants discussed the benefits they had received from respite away from the high acuity environment of the wards. All participants who referenced this were doctors, most consultants with other clinical or academic commitments and had concerns for others in the team who did not have this. “ How many? How is the reader to distinguish between a theme vs a single or two person anecdote? Page 8 In 29 “Some participants reported the use of practices to insulate themselves or others from the impact of their work. People reported self-identifying that they were unable to complete certain tasks (mask withdrawals and breaking bad news especially) and either delaying or delegating the task and senior decision makers also reported identifying staff who needed tasks reallocating. “ Please clarify what “practices to insulate” means. Page 10 line 45 Our data demonstrated significant personal impact on participants in keeping with quantitative studies[10] but has added greatly to this literature by identifying how working in the pandemic had this effect on staff supporting people with respiratory failure outside of ITU. Please elaborate on this statement. It is not readily apparent how this “added greatly” Page 9, In 42 Present throughout our data was the concept of repeated injury. It was not only that morally injurious events occurred, but that they occurred much more frequently than in usual circumstances. This was a significant finding present in every interview that appeared to exacerbate the impact felt by healthcare workers during the pandemic. I think this is the most significant finding in the paper. Please elaborate and compare/ contrast to the existing literature on other circumstances and outcomes from repeat injury What is the purpose of the drawing?
--	--

REVIEWER	Pratiwi, Ika Universitas Airlangga
REVIEW RETURNED	12-Mar-2022

GENERAL COMMENTS	interesting research but I have some input page 4 lines 5-10 please complete with reference sources page 4 lines 16-17, Are there any NARS guidelines or references used for clinicians at the research site. why is it a limited thing in the know? In which room in the hospital is NARS usually used? page 4 lines 21 and 22, does NARS have a lot of adverse effects on patients? why is it necessary to observe this at the clinician? page 4 lines 33-34, in appendix A what is the average length of work experience of each participant in the NARS administration? Which room in the hospital do you work in? this is related to the NARS experience later. page 5 lines 3-6, explain what is meant by "The team sought to identify areas of thematic consensus whilst recognizing the heterogeneous perspectives and experiences of the study participants"?. please relate it to the design you use and describe the interview questions you use that are relevant to the thematic identity intended here. It is necessary to explain the reference questions used in the interview. Does the group interview have no impact on the answers given by respondents to one another? how to prioritize originality of experience? page 7 line 13-15, what about the majority group? why take minority statements? page 8 lines 11-14, this statement can be a tendency because the medical considerations made need to be highlighted based on scientific logical rules page 10 lines 30-31, who are the "people" ? what is included in the interview? page 10 line 49, what does "ITU" stand for? page 10 line 53-54, is there any literature that supports this? is there any evidence to suggest that the statement is primarily about the loss of professional autonomy page 12 lines 18-23, which closing statement relates to your findings? page 12 lines 8-10, what are Poor staff continuity and inadequate staffing levels in your research? Is it related to workload or work experience? The background of working in a clinical setting has not been explained by the researcher, so this statement needs strong support many abbreviations that have not been given an explanation
---

VERSION 1 – AUTHOR RESPONSE

Reviewers Comment – Pearmain	Comments	Location of Actioned Outcome *location of outcome base on position in main document.
It may help to highlight the extent of the issue that both governments (Welsh government have issued a report in moral injury in healthcare workers) and professional bodies (BMA have issued report into moral injury) are concerned about moral injury to healthcare workers from COVID care.	Thank you, I have now included this in the discussion.	The concept of moral injury has become closely linked to the pandemic with statements of concern about the prevalence of moral injury from governments and professional bodies – [with ref to welsh gov and BMA statements] Page 10, Line(s) 1-4
In the introduction it would be helpful if the authors specified that the high mortality associated with CPAP is in patients not deemed likely to benefit from invasive mechanical ventilation. In those for escalation its role is limited to intubation avoidance. As such very few of the (generally) younger, fitter COVID-19 pneumonitis population die on CPAP, though I acknowledge managing them can be traumatic for staff in other ways.	I have clarified this in the intro and added more specific data on mortality amongst DNI patients.	NARS is used extensively in the treatment of respiratory failure due to covid-19. It is a first-line treatment for those (largely younger people) who would be suitable for escalation to intubation should this be required and for those (largely older people) for whom it constitutes the ceiling of treatment. The mortality rate is around 75% amongst those not considered appropriate for invasive ventilation and the treatment carries significant burden. A considerable number of these patients die either still using NARS or after its withdrawal; a procedure which represents a time of great uncertainty for both staff and patients. /span> Page 2, Line(s) 8-15
Whilst the study design appears appropriate I would recommend that the authors state why they felt a constructivist approach was the right one for this project apposed to other forms of thematic analysis.	I have now attempted to include a justification for the social constructivist approach without segueing into an unnecessarily complex qualitative theory background – as I know many readers would not be interested in this. I have further clarified our	A qualitative design drawing on a social constructionist perspective using semi-structured interviews. A social constructivist perspective was used to reflect the role that participants had in forming their own truth. Whilst each individual truth and the interpretation presented of

	inductive coding approach in the data analysis section.	these in our sample is valid, it may lack a universality when applied to other social contexts Page 2, Line(s) 29-32
The methods are generally clear and well described, however it is unclear why, or how the group interview differed from the individual interviews. This is important, as participants may influence each other and therefore accentuate themes etc.	It is known that less experienced professionals and nurses in particular are more likely to participate in interviews on a group basis rather than 1:1 and this was included as a participant preference. We acknowledge that the data may be qualitatively different in these two contexts but there is evidence that these approaches can provide complimentary and additive insights as well as broader diversity of participant engagement. In reality 3 people participated in one group interview. I have added additional details to the settings and participants section.	In order to enable the broadest diversity of participation of staff groups participants were invited to take part individually or in pairs or groups if preferred. Page 2-3, Line(s) 42-2
Within the description of participants (including supplementary table) I think it is important to be more specific about the breakdown of nurse colleagues, who will have very different roles and duties- this is important to confirm diverse sampling.	I have added this to the table and clarified in the settings and participant section	Appendix A
Within the methods "Data collection was completed when thematic saturation was achieved". Can the authors please confirm whether this was a pre-defined recruitment end-point, and how this was determined? If by a commonly accepted method then a reference could be used.	There is no one size answer to thematic saturation. We utilised the concept of exhausted inductive coding. I have added a reference to an overview article of different forms of saturation which includes descriptions of what I did by Uruhart, Given, Birks & Mills and Olshansky	A maximum of 37 participants were allowed for in the study design with premature closure to recruitment to occur on thematic saturation i.e. that no further new codes were added to subsequent transcripts at the analysis stage. This saturation end point prevents unnecessary data collection that does not add to the richness of the themes Page 3, Line(s) 2-6

Whilst some reflexivity is provided within the limitations in the discussion I feel this would be better stated more explicitly- perhaps as supplementary information alongside the specific roles they had. E.g who exactly conducted the interviews? Were both DW and LB present for all, did DW conduct some and LB some? Information about CF from a reflexive perspective may be appropriate, if they helped with interpretation/contextualizing the emerging themes for discussion.	I have clarified how the work was undertaken as well as show more information on the coding/thematic generation process.	The principle investigator, DW, is a doctor with extensive experience working in the NARS covid-19 wards with existing relationships with the participants. While this was a considerable advantage for recruitment and openness between participant and researcher, we recognise that this could have influenced the data gathered. CF is primarily an academic palliative care physician who spent no time in the acute trust during the pandemic. Under CF's supervision DW practiced a reflexivity-based approach to limit the impact of personal bias on data collection and analysis – this included keeping a reflexivity journal, regular supervision meetings and splitting data collection and analysis with a second researcher, LB. Researcher LB had no pre-existing relationships with the research participants and limited covid-19 exposure. LB carried out five of the interviews and analysed three independently. LB and DW jointly coded five of the transcripts and thematic generation took place in team meetings. Data was collected from only one hospital trust. Page 11, Line(s) 26-37
In the discussion I think it would be useful to highlight that there is no evidence/literature (that I could find/am aware) of NIV helping symptom control in COVID19 at all. It is then more obvious/reasonable to discuss NIV palliative role in COPD guidelines if the authors feel relevant (I'm not sure it can be extrapolated to COVID, but that is a matter of opinion not fact!).	I've added this to the discussion.	It should be noted that there is currently no literature to support the role of NARS as effective symptom relief in covid-19 and only limited evidence in COPD. Page 10, Line(s) 18-19
Having worked in the frontline I found the interviews emotive and	I agree this is potentially of concern.	Suggested text

brought me back to many of my own experiences. That is testament to how powerful this piece of work can be. It may be wise, if the editor decides to publish, to put a "trigger warning" statement early in the article. The quotations are likely to be the area of the article some readers may find difficult, so there is plenty of time to do this prior to then.		"Content Warning – quotes and topics within this article may be distressing to some readers."
---	--	--

Reviewers Comment – Whittle	Comments	Location of Actioned Outcome
I recommend further description of demographics of participants. How do they compare to staff in general? Why 15/21 female? – make appendix A into a table	Participants were selected based on purposive sampling related to job role. 48% of registered UK doctors, 88.3% of registered nurses and 76% of physiotherapists are female – our study is maybe slightly disproportionately female compared to national demographics but this is a moot point. Our study did not evaluate the differences of gender/ethnicity etc – we have not added this to the limitations section as we do not feel it is relevant to the nature of exploring moral injury in the same way it would be for quantifying it.	Reformatted appendix A.
Describe survey in more detail. Describe the interview process	For clarity I have now added the topic guide for the interview process to the article, contained in appendix B. As is good practice the topic guide was iteratively developed and this version incorporates such developments	Appendix B
Improve/ standardize abbreviations: MDT? NARS is more typically NIPPV	With regards to NARS Vs NIPPV – we took advice from a respiratory academic that the debate around whether NIV/NIPPV can be used to describe CPAP was contentious amongst some doctors (Kinneer, Non-invasive ventilation in acute respiratory failure, Thorax	

	2002;57:192-211 PubMed). To seek clarity we defined and used NARS as our acronym of choice to refer to a tight fitting pressure driven oxygen device. I have further clarified other acronyms as they arose.	
Was any validated PTSD screening survey used? Moral injury is often somewhat quantified using these and they seem appropriate here.	There is an obvious need to quantify the scale of moral injury but that was not the intention of this study. We set out to understand the experiences of staff and the emergent findings and led to the lens of moral injury as an explanatory framework. We did not a priori consider the study was about PTSD or moral injury.	
How severe was the impact to the participants? It was briefly noted that some required formal therapy, but was any attempt made to evaluate the extent of the injury?	The level of impact was not measured as part of this study although many participants shared description of the impact on themselves The findings provide understand of both the impacts themselves and how such impacts arose. we have noted the prevalence of psychological trauma including post-traumatic stress symptoms in the Introduction Certainly following this exploratory work there is scope to perform further vital research in this area.	
Further discuss the obvious significant limitations of small sample size, recall bias, etc	I have added in further clarification over the size of the sample. As well as addressing some of the limitations of the data with the scope of a social constructivist approach.	A maximum of 37 participants were allowed for in the study design with premature closure to recruitment to occur on thematic saturation i.e. that no further new codes were added to subsequent transcripts at the analysis stage. This saturation end point prevents unnecessary data collection that does not add to the richness of the themes Page 3, Line(s) 2-6 A social constructivist perspective was assumed to reflect the role that participants had in forming their truth. Whilst the truth presented is valid, it may lack a universality

		when applied to other social contexts Page 2, Line(s) 29-32 A degree of recall bias may have influenced data collection, especially as participants were asked to recall a memorable patient. However, this methodology allows significant in depth exploration of scenarios that are impactful to the participant, which was the aim of the research. Page 11, Line(s) 38-41
Page 8 line 18 “A minority of participants discussed the benefits they had received from respite away from the high acuity environment of the wards. All participants who referenced this were doctors, most consultants with other clinical or academic commitments and had concerns for others in the team who did not have this. “ How many? How is the reader to distinguish between a theme vs a single or two person anecdote?	The sample does not seek to be representative or reflect proportionality but to capture the diversity of experiences. It was purposive in its recruitment to try to achieve that. That said it can be useful to identify both commonality and variances in experiences. Due to the scales and sample design of qualitative research it is not meaningful to make statements about prevalence or distribution. Therefore, using numerical and quantifiable terms can be misleading. White, C., Woodfield, K., & Ritchie, J. (2003). Reporting and presenting qualitative data. Qualitative research practice: A guide for social science students and researchers, 2, 287-293	
Page 8 In 29 “Some participants reported the use of practices to insulate themselves or others from the impact of their work. People reported self-identifying that they were unable to complete certain tasks (mask withdrawals and breaking bad news especially) and either delaying or delegating the task and senior decision makers also reported identifying	Many thanks.	Changed ‘insulate’ to ‘protect’ Page 9, Line(s) 20

staff who needed tasks reallocating. “ Please clarify what “practices to insulate” means.		
Page 10 line 45 Our data demonstrated significant personal impact on participants in keeping with quantitative studies[10] but has added greatly to this literature by identifying how working in the pandemic had this effect on staff supporting people with respiratory failure outside of ITU. Please elaborate on this statement. It is not readily apparent how this “added greatly”	Many thanks, have removed greatly.	Our data demonstrated significant personal impact on participants in keeping with quantitative studies but has added to this literature by identifying how working in the pandemic had this effect on staff supporting people with respiratory failure outside of ITU. Page 9, Line(s) 31-34
Page 9, In 42 Present throughout our data was the concept of repeated injury. It was not only that morally injurious events occurred, but that they occurred much more frequently than in usual circumstances. This was a significant finding present in every interview that appeared to exacerbate the impact felt by healthcare workers during the pandemic. I think this is the most significant finding in the paper. Please elaborate and compare/ contrast to the existing literature on other circumstances and outcomes from repeat injury	The purpose of this study was to understand how the pandemic caused the psychological ill health present in existing literature. Repetitiveness was a significant finding but didn’t explain how seeing the same event over and over impacted participants. It was therefore not formulated into a theme. However, I have added it to the results introduction to underscore its significant undercurrent. I am not aware of studies quantifying the effect of repetitive injury. There certainly would be an avenue in exploring this further with quantifiable data to measure the impact of repeated moral injury and the advent of tools like MISS-HP (Mantri S, Lawson JM, Wang Z, Koenig HG. Identifying Moral Injury in Healthcare Professionals: The Moral Injury Symptom Scale-HP. J Relig Health. 2020;59(5):2323-2340. doi:10.1007/s10943-020-01065-w) to measure the scale	

	of impact are vital for this work. However, this is outside of the scope of this current project.	
What is the purpose of the drawing?	Removed	Removed

Reviewers Comment – Pratiwi	Comments	Location of Actioned Outcome
page 4 lines 5-10 please complete with reference sources	Many thanks, I have added three references to the NARS background.	
page 4 lines 16-17, Are there any NARS guidelines or references used for clinicians at the research site. why is it a limited thing in the know? In which room in the hospital is NARS usually used?	There is simply a lack of research in this area of care to inform guidelines. I have added a reference to a systematic review on dyspnoea in COPD to support this. Location of NARS care now added to settings and participants as per comments below.	Despite this, there is little data or guidance for clinicians caring for these patients at the end of life – perhaps because of the lack of research into symptom control in this area of care Page 2, Line(s) 16-18
page 4 lines 21 and 22, does NARS have a lot of adverse effects on patients? why is it necessary to observe this at the clinician?	This study was designed to explore the impact on clinicians. It was assumed this would be at the highest level where the most unwell/least likely to survive patients were. I have clarified this in the intro now. NARS has a significant treatment burden as is explored in the intro. I have not expanded on that as it feels outside the scope of this study. Further work into the impact on NARS on patients at end of life is clearly needed and will be the focus of future research from our team.	This study looks to explore the causes of this impact in the highest mortality area of covid-19 care – critically ill patients requiring NARS who were not suitable for further treatment escalation in invasive therapy. Page 2, Line(s) 21-23
page 4 lines 33-34, in appendix A what is the average length of work experience of each participant in the NARS administration? Which room in the hospital do you work in? this is related to the NARS experience later.	Many thanks. I have added this paragraph to the settings and participants section. I have not specified where participants worked as it allows to great an understanding of who they were (i.e. profession and location reveals who participants were within only a handful of potential participants.)	Participants had a range of experience with NARS. Four participants had less than five years' experience of working with NARS and 17 had more than five years' experience. Two of the consultant participants had worked in the founding group for introduction of NARS services to the acute hospital. Participants from the acute medicine hospital site worked within a medical unit dedicated to NARS care for the pandemic. Participants from the respiratory hospital worked

		either on the admissions unit or dedicated covid-19 NARS wards. Page 3, Line(s) 7-13
page 5 lines 3-6, explain what is meant by "The team sought to identify areas of thematic consensus whilst recognizing the heterogeneous perspectives and experiences of the study participants"?. please relate it to the design you use and describe the interview questions you use that are relevant to the thematic identity intended here. It is necessary to explain the reference questions used in the interview. Does the group interview have no impact on the answers given by respondents to one another? how to prioritize originality of experience?	Many thanks, I have simplified the sentence and then later linked that back to the role of semi-structured interviews in collecting that data. The topic guide for the interviews is now included in Appendix B. Added a note in results about the group interview. Originality and breadth of experience is captured with reference to unusual codes within themes i.e. the minority reported views.	. The team sought to identify areas of agreement and commonality between participants whilst recognising the range of perspectives and experiences described. These, sometimes even contradictory views, were crystallised into our final data analysis and added a profound richness to the data that was facilitated by the semi-structured interview format. Page 3, Line(s) 28-32 The group interview did not yield different codes or themes to the individual interviews and therefore will not be further identified in the results. Page 4, Line(s) 14-16
page 7 line 13-15, what about the majority group? why take minority statements?	Codes generated by majority views formed the main themes. Minority codes that still applied to the themes were included but identified as minority codes. This captures the breadth of experience without attempting to make quantifiable data from qualitative i.e. 3 people reported X. This study format does not lend itself to the production of quantitative data in that form.	
page 8 lines 11-14, this statement can be a tendency because the medical considerations made need to be highlighted based on scientific logical rules	I have further clarified how medical teams determined death was imminent with codes from the original data set. Interestingly while logical/scientific approaches are present (i.e. how much oxygen needs are increasing) there was also a tendency to rely on clinical experience that	It often became clear to clinicians and their teams that death was inevitable (based on oxygen requirements, ventilator dependency, work of breathing and clinical experience) before the family were ready to accept it. Page 6, Line(s) 23-26

	was more difficult for participants to verbalise.	
page 10 lines 30-31, who are the "people" ? what is included in the interview?	Many thanks	Incidents of 'people' replaced with participants for clarity.
page 10 line 49, what does "ITU" stand for	Many thanks	Replaced with intensive care units.
page 10 line 53-54, is there any literature that supports this? is there any evidence to suggest that the statement is primarily about the loss of professional autonomy	Replaced with 'perceived' loss of professional autonomy to reflect the subjective truth of the participants of the study.	This complex care environment often resulted in a perceived loss of professional autonomy – further confounding participants' moral actions. Page 9, Line(s) 37-38
page 12 lines 18-23, which closing statement relates to your findings?	Many thanks. To make more clear I have rewritten it. Preventing moral injury by making staff feel they have provided good care means giving them to tools and understanding to provide to good care. And, therefore, further research in NARS in end of life care is important.	A peaceful and holistically provided for patient death was often viewed by participants as compensation for a tumultuous journey. The role NARS may have in a peaceful death as symptom control for breathlessness is an understudied phenomenon. Further research on the role of NARS in end-of-life care will be important to continue to improve patient care and support our staff in facilitating good end-of-life care. Page 11, Line(s) 20-24
page 12 lines 8-10, what are Poor staff continuity and inadequate staffing levels in your research? Is it related to workload or work experience? The background of working in a clinical setting has not been explained by the researcher, so this statement needs strong support	Objective data about staffing and continuity is outside the scope of this study. This is about the subjective truth of the participants. However, I agree it is important to be clear and so I have edited the statement to include the word 'perceived'. A truth to the managing department or to myself, as a clinician, may be that staffing was adequate and continuity good.	Perceived poor staff continuity and inadequate staffing levels exacerbate issues within this context but may not be easily addressed Page 11, Line(s) 11-13
many abbreviations that have not been given an explanation	Apologies, many got lost in the editing process. Now restored with explanations.	Various.

VERSION 2 – REVIEW

REVIEWER	Pearmain, Laurence Wellcome Trust Centre for Cell Matrix Research
REVIEW RETURNED	28-Apr-2022

GENERAL COMMENTS	All comments from previous version addressed satisfactorily, with clearer presentation of methods especially. An engaging paper on an important topic- thank you for sharing it with me.
---

REVIEWER	Whittle, Jessica S. University of Tennessee
REVIEW RETURNED	28-Apr-2022

GENERAL COMMENTS	Thank you for an excellent response to reviewers
--