# Supplementary material for: Impact on staff of providing non-invasive advanced respiratory support during the COVID-19 pandemic: a qualitative study in an acute hospital
Source: BMJ Open. 2022 Jun 1;12(6):e060674. doi: 10.1136/bmjopen-2021-060674 (PMC9160594; doi:10.1136/bmjopen-2021-060674)
Supplement: Supplementary data [file bmjopen-2021-060674supp001.pdf]

Appendix A

Demographic Traits	Female	Male
Ward Nurses	1	0
Specialist Nurses (Palliative Care)	5	0
Junior Doctors (registrar or below)	3	1
Consultants (Acute/General Med)	1	2
Consultants (Respiratory)	2	1
Consultants (Palliative Medicine)	2	1
Physiotherapists	1	1
Total	15	6
