# Supplementary material for: Impact on staff of providing non-invasive advanced respiratory support during the COVID-19 pandemic: a qualitative study in an acute hospital
Source: BMJ Open. 2022 Jun 1;12(6):e060674. doi: 10.1136/bmjopen-2021-060674 (PMC9160594; doi:10.1136/bmjopen-2021-060674)
Supplement: Author's manuscript [file bmjopen-2021-060674.draft_revisions.pdf]

BMJ Open is committed to open peer review. As part of this commitment we make the peer review history of every article we publish publicly available.

When an article is published we post the peer reviewers' comments and the authors' responses online. We also post the versions of the paper that were used during peer review. These are the versions that the peer review comments apply to.

The versions of the paper that follow are the versions that were submitted during the peer review process. They are not the versions of record or the final published versions. They should not be cited or distributed as the published version of this manuscript.

BMJ Open is an open access journal and the full, final, typeset and author-corrected version of record of the manuscript is available on our site with no access controls, subscription charges or pay-per-view fees (http://bmjopen.bmj.com).

If you have any questions on BMJ Open's open peer review process please email info.bmjopen@bmj.com

BMJ Open

The Impact on Staff of Providing Non-Invasive Advanced Respiratory Support During the Covid-19 Pandemic– A Qualitative Study in an Acute Hospital

Journal:	*BMJ Open*
Manuscript ID	bmjopen-2021-060674
Article Type:	Original research
Date Submitted by the Author:	29-Dec-2021

Complete List of Authors:	Wenzel, David; University of Leicester<br>Bleazard, Lucy; University of London<br>Wilson, Eleanor; University of Nottingham, Faculty of Medicine & Health Sciences<br>Faull, Christina; LOROS Hospice

Keywords:	COVID-19, QUALITATIVE RESEARCH, Public health < INFECTIOUS DISEASES

SCHOLARONE™
Manuscripts

I, the Submitting Author has the right to grant and does grant on behalf of all authors of the Work (as defined in the below author licence), an exclusive licence and/or a non-exclusive licence for contributions from authors who are: i) UK Crown employees; ii) where BMJ has agreed a CC-BY licence shall apply, and/or iii) in accordance with the terms applicable for US Federal Government officers or employees acting as part of their official duties; on a worldwide, perpetual, irrevocable, royalty-free basis to BMJ Publishing Group Ltd ("BMJ") its licensees and where the relevant Journal is co-owned by BMJ to the co-owners of the Journal, to publish the Work in this journal and any other BMJ products and to exploit all rights, as set out in our licence.

The Submitting Author accepts and understands that any supply made under these terms is made by BMJ to the Submitting Author unless you are acting as an employee on behalf of your employer or a postgraduate student of an affiliated institution which is paying any applicable article publishing charge ("APC") for Open Access articles. Where the Submitting Author wishes to make the Work available on an Open Access basis (and intends to pay the relevant APC), the terms of reuse of such Open Access shall be governed by a Creative Commons licence – details of these licences and which Creative Commons licence will apply to this Work are set out in our licence referred to above.

Other than as permitted in any relevant BMJ Author's Self Archiving Policies, I confirm this Work has not been accepted for publication elsewhere, is not being considered for publication elsewhere and does not duplicate material already published. I confirm all authors consent to publication of this Work and authorise the granting of this licence.

For peer review only

The Impact on Staff of Providing Non-Invasive Advanced Respiratory Support During the Covid-19 Pandemic– A Qualitative Study in an Acute Hospital

For peer review only

Correspondence to: Dr David Wenzel, LOROS Hospice, Groby Road, Leicester, LE3 9QE
David.wenzel@nhs.net

Dr Lucy Bleazard, School of Medicine, University of Leicester, Leicester, UK

Dr Eleanor Wilson, School of Health Sciences, University of Nottingham, Nottingham, UK

Professor Christina Faull, Palliative Care, LOROS Hospice, Leicester, UK

Main Text Word Count: 4299

Objectives: To explore the experiences of healthcare workers of providing Non-Invasive Advanced Respiratory Support (NARS) to critically unwell patients with covid-19.

Design: A qualitative study drawing on a social constructionist perspective using thematic analysis of semi-structured interviews.

Setting: A single acute UK NHS Trust across two hospital sites.

Participants: Multidisciplinary team members in acute, respiratory and palliative medicine.

Results: 21 nurses, doctors (juniors and consultants) and physiotherapists described the provision of NARS to critically unwell covid-19 patients as extremely challenging. The main themes were of feeling ill prepared and unsupported, a need to balance complex moral actions, a sense of duty to patients and their families. The impact on staff was profound and findings are discussed via a lens of moral injury. Injurious events included staff feeling they had acted in a way that caused harm, failed to prevent harm or had been let down by seniors or the Trust. Participants identified factors that mitigated adverse impact.

Conclusions: Although many of the issues described by participants are likely immutable components of healthcare in a pandemic there were several important protective factors that emerged from the data. Experience, debriefing and breaks from covid-19 wards were valuable to participants and successfully achieving a peaceful death for the patient was often viewed as compensation for a difficult journey. These protective factors may provide modelling for future education and support services to help prevent moral injury – or aide in its recovery.

Registration: registered on the Open Science Framework 10.17605/OSF.IO/XB8YH

Strengths and Limitations

- Single Centre Study
- The lead author had pre-existing relationships with study participants
- The lead author, under strict supervision, practiced a reflexivity based approach to data collection and analysis with objectivity benchmarking from the second author.

For peer review only

INTRODUCTION

Non-invasive advanced respiratory support (NARS), including continuous positive airway pressure (CPAP) or bilevel positive airway pressure (BiPAP, sometimes referred to as simply non-invasive ventilation (NIV)) is an essential component of modern clinical practice. It is used for the treatment of critically ill patients with a range of common conditions including COPD, pulmonary oedema and, more recently, covid-19.

NARS is used extensively in the treatment of covid-19, despite a high mortality rate and significant treatment burden[1]. A considerable number of patients die either still using NARS or after its withdrawal; a procedure which represents a time of great uncertainty for both staff and patients[2]. Despite this, there is little data or guidance for clinicians caring for these patients at the end of life.

Quantitative studies of the impact of covid-19 on healthcare workers have shown high levels of post-traumatic stress symptoms[3], notably greater than in other infectious disease outbreaks[4]. This study looks to explore the causes of this impact in the context of NARS and covid-19. There is a paucity of data both on how best to provide care for these patients as they die and what impact this has on those who care for them.

METHODS

Design

A qualitative design drawing on a social constructionist perspective using semi-structured interviews[5,6]. Initially participants described a memorable patient who had used NARS and died and an iteratively developed topic guide facilitated further exploration of issues, practices and experiences[6]. Evaluated before publication against the SRQR reporting guidelines[7].

Settings and Participants

Doctors, nurses and physiotherapists from acute medicine, respiratory medicine and palliative care were recruited using a purposive sampling approach from wards involved in the delivery of NARS in two hospital sites of an acute NHS Trust in England. See appendix A for full details of participants.

Patient and Public Involvement

No patient or public involvement was sought in the creation of this study as it focused solely on the impact of healthcare workers and contained no patient or public participation in data collection. Results from this study will be disseminated to participants via internal email.

Data Collection

19 interviews of 21 participants were undertaken by telephone (n=2), Microsoft Teams (n=16) or face to face interview (n=1) audio recorded (encrypted voice recorder, Olympus DS-3500), and transcribed verbatim. Three participants opted for a group interview.

Data Analysis

Thematic development was contemporaneous but dynamic and in parallel with data collection based on a principles of constant comparison[8], supported by MAXQDA Plus 2020 (V20.4.0). Open coding and development of the initial coding frame was undertaken

For peer review only - http://bmjopen.bmj.com/site/about/guidelines.xhtml

by DW and LB with iterative group discussion. The team sought to identify areas of thematic consensus whilst recognising the heterogeneous perspectives and experiences of the study participants. These, sometimes even contradictory views, were crystalised into our final data analysis and added a profound richness to the data[9]. Data collection was completed when thematic saturation was achieved.

Ethics
NHS Research Ethics Committee review was not required as the study involved only NHS staff. However, local sponsorship was provided by the University Hospitals of Leicester (EDGE ID 138908).

RESULTS

In all interviews participants discussed both the high level and the harmful consequences of the emotional, physical and psychological impact levied upon them and their colleagues by the covid-19 pandemic.

> *"… you know when you wear the FFP3[1] masks? I can smell that smell [and] it takes me back to the first lady that I'd been involved in a CPAP withdrawal for and I just remember it…" (Specialist Palliative Care Nurse)*

> *"… it massively impacted me emotionally… I got really anxious. I wasn't really sleeping. I wasn't eating much. I had a bit of talking therapy" (Junior Doctor)*

> *"I was emotionally broken. I remember crying at the end of that weekend. I was, I was like 'Oh my God' and I had to, I had to actually reach out to my consultant colleagues to say, 'I feel broken' and they were like, 'that's normal'." (Acute Medicine Consultant)*

Five themes emerged from the data that describe the causes of such impact and factors that may protect against harmful consequences.

Ill Prepared and Unsupported
Participants expressed concern over educational needs that were not met prior to deployment. Whilst it was noted that a lack of education around end-of-life care and NARS, including ethics and clinical reasoning, predated the pandemic, the effects of this were compounded by covid-19. Participants relied on informal teaching from colleagues and other sources (such as popular literature) to make up for this shortfall.

> *"Actually, I learn most of what I know about death and dying from working with my palliative care colleagues" (Physiotherapist)*

There was a perceived lack of opportunity for debriefing with most reflection after traumatic events being informal between colleagues. There was often a feeling that more

[1] FFP3 – Filtering Face Pieces 3, a tight-fitting filtration mask used as part of personal protective equipment during exposure to aerosol generating procedures.

For peer review only - http://bmjopen.bmj.com/site/about/guidelines.xhtml

For peer review only

could be done at an organisational level to support staff in areas with high levels of NARS covid-19 care.

> *"I'm not sure we got a lot from the Trust... I think there was very little formal support. I think that compared to some areas we did very badly on getting debriefs and things organized." (Acute Medicine Consultant)*

Being short of staff and poor continuity of care were referenced by many participants as having negative impacts on their ability to provide care.

Moral Actions

Participants spoke often of actions undertaken during the pandemic that had a moral underpinning; acts that balance burden and benefit for the patient, acts that they were compelled or coerced to perform; and acts they were unable to complete.

NARS was a treatment regarded as unlikely to be successful with significant treatment burden and was often felt to be started due to a lack of other options, rather than as an act in keeping with the patient's best interests. Some participants reflected that starting NARS was a way for staff to cope personally with so many deaths,

> *"You've forced a treatment that is unpleasant on somebody... And sometimes it feels like you're assaulting them... [because] people just desperately don't want another person to die. But sometimes there's just the feeling of helplessness - I think that they're feeling that [starting CPAP] is a way that they can manage that feeling of the tsunami of dying." (Palliative care consultant)*

The decision to continue NARS weighed on participants in a similar fashion and even decisions to change interface made interviewees feel complicit in this burdensome treatment.

> *"The mask wasn't giving her much symptom relief, it wasn't giving her much support with oxygenation. So we went for the hood ... to see if it made a difference A) to her symptoms and B) to her clinical condition ... It didn't make a difference." (Physiotherapist)*

Over half of participants reported concerns they were giving patients and their families false hope, because the chance of recovering was minimal.

> *"Very often it's just the fact that the family are holding onto that chink of hope that something is dramatically going to turn around and the patient will improve. And you know it's not going to happen 'cause you've been there many times before." (Respiratory consultant)*

Communicating the poor prognosis and the imminence of death weighed on participants' minds:

> *"... you're looking someone in their eyes and telling them that they're dying. So that's the difficulty." (Respiratory Consultant)*

If CPAP treatment became too burdensome, with no chance of recovery, it was often withdrawn on the advice of senior clinicians or at the request of the patient themselves. The

For peer review only - http://bmjopen.bmj.com/site/about/guidelines.xhtml

process of withdrawal was often described as traumatising to staff. Participants felt a responsibility to withdraw the mask and enable the patient to die as peacefully and symptom free as possible but anticipating a patient's needs and to manage any distress was challenging.

Healthcare workers had a range of feelings after withdrawals. Some reported an immense sadness. The removal of the mask emphasised the teams' perceived failure to save that person's life. Some saw the withdrawal as an opportunity to create peace and a natural death, regarding it as essential for holistic palliative care. A minority of participants reported feeling responsible for causing the death by removing the mask - but most participants discussed knowing that others felt this way, even if they themselves did not.

> *"… being that person who takes it off, it can sometimes feel like you're the person ending that patient's life" (Physiotherapist)*

The visiting restrictions participants described varied throughout the pandemic. At times only patients dying *and* not on NARS were allowed end of life visitation. The recognition of death was often felt to be delayed and late in the dying process which further complicated visiting. Participants spoke extensively about restricting visitation from relatives during the patients' admission and especially when they were dying. This had a profound impact on some.

> *"But just to keep a family member away from their dying relative is probably one of the hardest things I've ever had to do." (Acute Medicine Consultant)*

Loss of Professional Autonomy

Autonomy was often felt to have been lost at the expense of managing the patients' families' expectations and readiness for the death of their loved one. It often became clear to clinicians and their teams that death was inevitable before the family were ready to accept it. For patients who deteriorated despite NARS its withdrawal was generally seen as the logical/ethical clinical decision due to its progressive loss of symptomatic relief and increasing treatment burden (claustrophobia, impact on communication, breathlessness, anxiety). There were also professional concerns around prolonging and medicalising the dying process. These approaches were sometimes in tension with family views about the value of continuing NARS:

> *"I think the family were more so reluctant themselves to have the CPAP mask removed. So for them I guess it provided them a sense of comfort that at least everything was done till the very end." (Ward Nurse)*

A huge amount of communication was dedicated to ensuring that families understood their loved one's condition and were enabled to accept the clinical recommendations. If families could not concur or be helped to be ready prior to their loved one's death an extreme amount of distress was reported.

> *"… they wanted his wife to be able to see him over Facetime and they had the phone with the wife, in his face, while he was dying and gasping… they had removed the CPAP…. I can remember this distinct memory of wailing and screaming and I'm just*

For peer review only - http://bmjopen.bmj.com/site/about/guidelines.xhtml

> *sitting there trying to make it stop and the patient died, he died, whilst the wife was in [his] face because they wanted to see his face…" (Specialist Palliative Care Nurse)*

Even when families were accepting of the clinical decisions being made, staff often felt their range of options were limited due to inheriting decisions from the previous shifts or earlier in the chain of care.

> *"In my own mind I would think, 'why have they been put on CPAP?'. …But there wasn't somebody I could have a disagreement with because the decision had been made 2, 3, 5 days earlier." (Acute Medicine Consultant)*

Whilst respecting senior decision-makers and the complexity of their role, there were many references to staff being involved in clinical decisions they would not make themselves and the impact that this had on them.

> *"I'm probably more distressed by the people who I felt I supported a decision, or was coerced it felt like, into helping them have CPAP when I thought they were dying in the next few hours." (Palliative Care Consultant)*

This was often due to the uncertainty of the clinical situation and at times led to conflict within the MDT about the appropriateness of continuing therapy and prolonging or medicalising death. Disagreements were often reported as not openly discussed and the data suggest that this resulted in little awareness from senior decision makers.

> *"It's quite nice getting their opinions but I wouldn't necessarily do what they wanted if it wasn't what I wanted to do." (Respiratory Consultant)*

> *"I know from a nursing perspective a lot of the nurses felt uncomfortable… I feel like a medical decision is probably very different to how the nurses felt." (Ward Nurse)*

Participants frequently described their difficulty in controlling the severe symptoms that were caused by the patients' illness. Their efforts were further complicated by the impact of PPE requirements.

> *"And became massively tachypnoeic and was trying to sit up at that point to get his breath, which was horrific. So, and the difficulty being…you've got to put on [PPE] to get in there." (Specialist Palliative Care Nurse)*

These symptoms often occurred in patients with little history of ill health and in the context of rapid clinical decline. This made them more poignant to the staff that witnessed them.

Duty

Exposure to pandemic experiences was often viewed in the context of a sense of duty, to patients and their families. The moral actions participants undertook were viewed in the context of a deep sense of dedication to doing the 'right' thing and a responsibility for how families would perceive the death of their loved ones

> *"I think it's only human to try and avoid the difficult decisions but I think it's important that we have a duty of care where we try and address things that are difficult." (Junior Doctor)*

For peer review only - http://bmjopen.bmj.com/site/about/guidelines.xhtml

> *"… you're guiding this experience for someone. And how are they going to look back on it? And are they going to look back on it and go, 'Yeah, that was OK.' Or are they going to look back and say, 'that was a disaster'." (Palliative Care Consultant)*

This sense of duty led to actions by staff significantly beyond their usual role; acting as substitute family; partaking in end-of-life religious rituals; sitting in full PPE while a patient died; or taking part in end-of-life conversations that healthcare workers are rarely party to:

> *"… you effectively become, the nursing staff and the medical team, effectively become the surrogate family" (Junior Doctor)*

> *"But prior to covid you wouldn't sit and listen to somebody else tell their Mum all their things that they want to in the minutes before she dies… you wouldn't witness all of that." (Palliative Care Consultant)*

Ultimately, it appeared this sense of duty was often the driving factor for perseverance from otherwise expended staff:

> *"It was very much a 'we've just got to get on with this, we've just gotta get through this as best we can, remember that all of these patients are relying on us to make them comfortable'." (Palliative Care Nurse)*

Protective Factors

Participants discussed successes they had during the pandemic and the factors that facilitated them. These seemingly buoyed their perceptions of their teams' actions and led to greater satisfaction with care provision, providing some protection from the ongoing strains of NARS care in the pandemic.

Dying peacefully was often referenced as a compensation for the overall nature of a patient's clinical journey. Ensuring minimal symptoms, family presence and every avenue of care had been explored gave participants a sense of accomplishment. The earlier clinical decisions were made, and the more clearly they were communicated, the more associated they were with better patient care:

> *"I had a case on [a ward] who went on and did a night on CPAP and didn't tolerate it and they stopped it overnight at two o'clock in the morning. And actually, he then went home and died at home* [which was his wish]. *But my point more is that they sort of clocked... They didn't persevere. They clocked early that this wasn't going well. They got out." (Palliative Care Consultant)*

Higher levels of clinical experience were associated with increased resilience and surety around clinical decision making.

> *"It's a decision that you make. I can't predict the future anymore than anybody else can. But it's a case of you have to make a decision. And that's part and parcel of having been a consultant and having the luxury of experience." (Acute Medicine Consultant)*

For peer review only - http://bmjopen.bmj.com/site/about/guidelines.xhtml

Regardless of whether death was peaceful or not staff spoke highly of their colleagues' support afterwards. This support was often informal, based on local team dynamics and often individuals, but felt to be a valuable part of the recovery process:

> *"We often talk to [name]. She is really good at asking people how they are. People like talking to her, she's very sympathetic. And then I know the matron is very much on hand to talk to the nurses" (Respiratory Consultant)*

> *"Some of the senior nurses were just fabulous, fabulous people, and we were being massively supported by them as well, as much as they would say we supported them. There were days when I was on my knees going "I just can't come back to this," you know." (Specialist Palliative Care Nurse)*

A minority of participants discussed the benefits they had received from respite away from the high acuity environment of the wards. All participants who referenced this were doctors, most consultants with other clinical or academic commitments and had concerns for others in the team who did not have this.

> *"It doesn't seem to have a particular effect on me but maybe I just had… less involvement over the last six months and that's allowed me to sort of lick my wounds and feel better for it." (Acute Medicine Consultant)*

Some participants reported the use of practices to insulate themselves or others from the impact of their work. People reported self-identifying that they were unable to complete certain tasks (mask withdrawals and breaking bad news especially) and either delaying or delegating the task and senior decision makers also reported identifying staff who needed tasks reallocating.

> *"That's not always possible, but we also try to recognize that in our team… when we've done several difficult ones in a day, or in a few days, that actually you may not be the right person to do the next one a few hours later. Because you might not have enough of yourself to give." (Palliative Care Consultant)*

DISCUSSION

Our data demonstrated significant personal impact on participants in keeping with quantitative studies[10] but has added greatly to this literature by identifying how working in the pandemic had this effect on staff supporting people with respiratory failure outside of ITU. Participants were forced by circumstance to engage in complex moral actions balancing burden and benefit while treating patients with severe symptoms. This impact was compounded by the overwhelming volume of patients and the duty staff felt to help them and their families. This complex care environment often resulted in a relative loss of professional autonomy – further confounding participants' moral actions.

Analysis revealed significant congruence between our data and the existing canon of knowledge around the theory of moral injury. Moral injury refers to the impact felt when a person experiences events not in keeping with their own moral code, or conscience. These events are often subdivided into acts of commission, omission and betrayal[11]. Many of the actions undertaken by participants were moral actions that balanced cost or burden and

For peer review only - http://bmjopen.bmj.com/site/about/guidelines.xhtml

benefit: restricting visiting, initiating high burden treatment and withdrawing treatment.

Being forced to balance a patient's desire and right to see their family before they die and the safety of their family and society may result in a morally 'correct' action, but the process of enacting this and the witnessed distress it causes is injurious to the practitioner. This is consistent with an act of commission, an action a person is compelled to undertake by seniors or the environment of care[12].

The value of NARS was a balance of burdensome treatment and of hope of possible recovery and symptom relief, conceptually the opposing energies of an anchor and a buoy (see Appendix B in artwork by Nadine Coffey). An inability to stop death, to control symptoms adequately or act in the best interest of the patient weighed heavily on participants. These represent acts of omission, acts that are not undertaken by healthcare workers due to the environment in which they are working, and this led participants to feel as if their care was lacking.

Some of the most challenging experiences participants described were around the loss of autonomous decision making. Feeling coerced into forcing treatment on a patient by colleagues or feeling unable to withdraw due to family readiness impacted participants who then felt complicit in the suffering that ensued. This loss of autonomy represents both an act of omission and an act of commission.

Participants expressed concern over educational needs that had not been met prior to deployment to covid-19 wards as well as a lack of formal debriefing opportunity after challenging cases. This represents an act of betrayal, where a trusted other creates exposure to injurious events – often through lack of preparation or support[13]. However, in contrast with existing literature[14], no references were made by participants to personal protective equipment availability and only one participant described concerns over level of exposure and the risk of catching covid-19. Betrayal as an origin of moral injury was less prevalent across interviews than reflections on commission and omission.

Literature around moral injury describes those affected as 'witnesses'[12] to suffering. Our data suggested that the impact from injurious events was facilitated by the participants sense of ownership over the traumas they witnessed. The duty that they felt to provide good care and support to families was key in the mechanism of their suffering. This sense of 'ownership' should be considered in future works examining moral injury in healthcare workers.

Present throughout our data was the concept of repeated injury. It was not only that morally injurious events occurred, but that they occurred much more frequently than in usual circumstances. This was a significant finding present in every interview that appeared to exacerbate the impact felt by healthcare workers during the pandemic.

Moral Injury represents a wide spectrum of impact, from self-limiting to chronically debilitating and requiring professional treatment[15]. This spectrum was present in our data. Some participants were unaffected, some had persistent symptoms and a minority spoke about requiring formal treatment to recover (psychological therapy).

There was great openness from participants to discuss the impact that providing end of life care in a pandemic had on them. This in and of itself may be indicative of the lack of formal structures in place to help staff recover. Lack of support in recovery was described frequently but there was no clear consensus over what would be most helpful in this regard. The impact on staff of low impact/high burden treatments is rarely considered in the literature but may need to form a key part of any support packages used in the future. The low level efficacy of NARS was anecdotally apparent to staff but not apparent in formal

For peer review only - http://bmjopen.bmj.com/site/about/guidelines.xhtml

evidence throughout much of the pandemic and only recent papers formally question its use in 'do not intubate' patients[1].

Our findings identify several issues that may form key components of preventative strategy including: education around NARS use and withdrawal[16], the ethical reasoning around NARS and how we prepare staff for patient death. Poor staff continuity and inadequate staffing levels exacerbate issues within this context but may not be easily fixed.

A great many of the components of moral injury to staff were, and remain, seemingly unavoidable. As the patients suffered, so did their caregivers. This may be a component of pandemic healthcare[17,18]. However, how we prepare, support and aide recovery warrants further investigation. Participants described several coping mechanisms, debriefing requirements and post injury recovery techniques that could aide with workforce planning moving forward.

Furthermore, the effectiveness of NARS in management of breathlessness is an understudied phenomenon[19] and to what extent it supports symptom management when a patient is dying is not clear. There is acknowledgement in acute NIV guidelines for COPD that good quality palliation can take place with a mask in situ if preferred[20] but how to provide this care in the context of how burdensome NIV is[21], is not explored.

LIMITATIONS

The principle investigator, DW, is a doctor with extensive experience working in the NARS covid-19 wards with existing relationships with the participants. While this was a considerable advantage for recruitment and openness between participant and researcher, we recognise that this could have influenced the data gathered. Under CF's supervision DW practiced a reflexivity-based approach to the research to limit the impact of personal bias on data collection and analysis. Researcher LB had no pre-existing relationships with the research participants and limited covid-19 exposure. Her interviews and data analysis provided an objectivity 'benchmark' to monitor for bias in DW's work. Data was collected from only one hospital trust.

CONCLUSION

This study identified the considerable impacts on health care staff caring for patients who required NARS and died due to covid-19. It has identified the multifactorial origins of distress and harm to staff and explores this within the context of the duty that staff felt towards their patients which augments the current understanding of moral injury.

A great many of the factors that impacted on staff are immutable and unavoidable components of severe respiratory disease. Being unable to reverse an illness process, witnessing distress and breaking bad news are regrettable essentials of this work. However, the role of staff as active agents of distress – restricting visitation, starting high burden treatments, witnessing family distress and a sense of involvement in prolonging suffering or causing death may require further reflection should the pandemic worsen, or a new one occurs. Importantly the study was able to identify several protective factors that may prevent, reduce and aide recovery from injurious events. Understanding how to protect and minimise psychological injury to our workforce in these impactful circumstances will be essential in safeguarding the future of healthcare provision.

For peer review only - http://bmjopen.bmj.com/site/about/guidelines.xhtml

Transparency Declaration[22]
The lead author, Dr David Wenzel, affirms that this manuscript is an honest, accurate, and transparent account of the study being reported; that no important aspects of the study have been omitted; and that any discrepancies from the study as planned (and, if relevant, registered) have been explained.

Contributorship Statement
Contributors: DW and CF conceptualised and developed the project. The protocol was authored by DW, who is the guarantor, and reviewed/edited by CF. Data collection tools were designed by all authors. Data collection was performed principally by DW supported by LB. Database creation was principally by DW, supported by LB. Data analysis was performed principally by DW with support from LB, EW and CF (contribution level in order). Thematic analysis was performed equally by DW and LB with further contribution from CF and EW. The primary report was authored by DW with editing and review by EW, CF and LB (contribution level in order).

Competing Interests
We have read and understood the BMJ policy on declaration of interests and declare the following interests: None.

Funding
The lead author's current posting, academic clinical fellow, is a funded role by the National Institute of Health Research (NIHR). The NIHR had no input in study creation, design or reporting. Funding award reference ACF-2019-11-007.

Data Sharing Statement
Data are available upon reasonable request. All raw materials including de-identified raw data, coding and thematic analysis can be obtained by contacting lead author Dr David Wenzel via email at David.wenzel@nhs.net.

References
1 Bradley P, Wilson J, Taylor R, et al. Conventional oxygen therapy versus CPAP as a ceiling of care in ward-based patients with COVID-19: a multi-centre cohort evaluation, *EClinicalMedicine,* 2021;10:40;101122

2 Hough A, Dell GD, Blaber M, et al. Beyond the mask: a multidisciplinary reflection on palliating patients with COVID-19 receiving continuous positive airway pressure ventilation, *Int J Palliat Nurs,* 2020;10:2;26(7):336-340

3 Mosheva M, Gross R, Hertz-Palmor N, et al. The association between witnessing patient death and mental health outcomes in frontline COVID-19 healthcare workers. *Depress Anxiety* 2021;4:38(4):468-479.

4 Lamb D, Gnanapragasam S, Greenberg N, et al. Psychosocial impact of the COVID-19 pandemic on 4378 UK healthcare workers and ancillary staff: initial baseline data from a cohort study collected during the first wave of the pandemic. *Occupational and Environmental Medicine,* 2021;6:78:801-808.

For peer review only - http://bmjopen.bmj.com/site/about/guidelines.xhtml

5 Charmaz K. Constructing Grounded Theory; A Practical Guide Through Qualitative Analysis, London, Sage Publications, 2006: 125-131.

6 Rubin HJ, Rubin IS. Qualitative Interviewing: The art of hearing data, London, Sage Publications, 2005: 64-78

7 O'Brien BC, Harris IB, Beckman TJ, Reed DA, Cook DA. Standards for reporting qualitative research: a synthesis of recommendations. *Acad Med*. 2014;89(9):1245-1251

8 Charmaz K. Constructing Grounded Theory; A Practical Guide Through Qualitative Analysis, London, Sage Publications, 2006: 42-71.

9 Barbour R S. Checklists for improving rigour in qualitative research: a case of the tail wagging the dog? *BMJ,* 2001; 322 :1115

10 Hines SE, Chin KH, Levine AR, Wickwire EM. Initiation of a survey of healthcare worker distress and moral injury at the onset of the COVID-19 surge. *Am J Ind Med,* 2020;63(9):830-833

11 Ford, EW. Stress, Burnout, and Moral Injury: The State of the Healthcare Workforce, *J Healthc Manag*. 2019;5-6:64;3;125-127

12 Cartolovni A, Stolt M, Scott PA, et al. Moral injury in healthcare professionals: A scoping review and discussion. *Nurs Ethics*. 2021;1:28(5);590-602.

13 Shay J. Moral Injury. *Psychoanal Psychol*. 2014:31;2;182-191

14 French L, Hanna P, Huckle C. “If I die, they do not care”: U.K. National Health Service staff experiences of betrayal-based moral injury during COVID-19. *Psychol Trauma*. [preprint] December 07, 2021 https://doi.org/10.1037/tra0001134

15 Williamson V, Murphy D, Phelps A, et al. Moral injury: the effect on mental health and implications for treatment. *Lancet Psychiatry*. 2021;3:8(6), 453–455.

16 Faull C, Phelps K, Regen E, et al. Withdrawal of Ventilation at the patient’s request in MND: Doctors views on the ethical challenges involved. *BMJ Support Palliat Care*. 2014;4:A39

17 Bernstein M, Hawryluck L. Challenging beliefs and ethical concepts: the collateral damage of SARS. *Crit Care*. 2003;5:7;269

18 Leong IY, Lee AO. The challenge of providing holistic care in a viral epidemic: opportunities for palliative care. *Palliat Med*. 2004:18:12-18

19 Smith TA, Davidson PM, Jenkins CR, et al. Life behind the mask: the patient experience of NIV. *Lancet Respir Med* 2015;3:8–10.

20 Craig D, Steve B, Mark E, et al. BTS/ICS Guidelines for the Ventilatory Management of Acute Hypercapnic Respiratory Failure in Adults, *Thorax* 2016;4:71 Suppl 2

21 Smith TA, Agar M, Jenkins CR, et al. Experience of acute noninvasive ventilation—insights from ‘Behind the Mask’: a qualitative study, *BMJ Support Palliat Care*, 2019;9:e11.

For peer review only - http://bmjopen.bmj.com/site/about/guidelines.xhtml

22 Altman D G, Moher D. Declaration of transparency for each research article. *BMJ*. 2013; 347:f4796

For peer review only

For peer review only - http://bmjopen.bmj.com/site/about/guidelines.xhtml

Appendix A

Demographic Traits	Number of Participants
Male	6
Female	15
Nursing team (including specialist nurses)	6
Junior Doctors (registrar or below)	4
Consultants (Acute/General Med)	3
Consultants (Respiratory)	3
Consultants (Palliative Medicine)	3
Physiotherapists	2
Total Participants	21

For peer review only

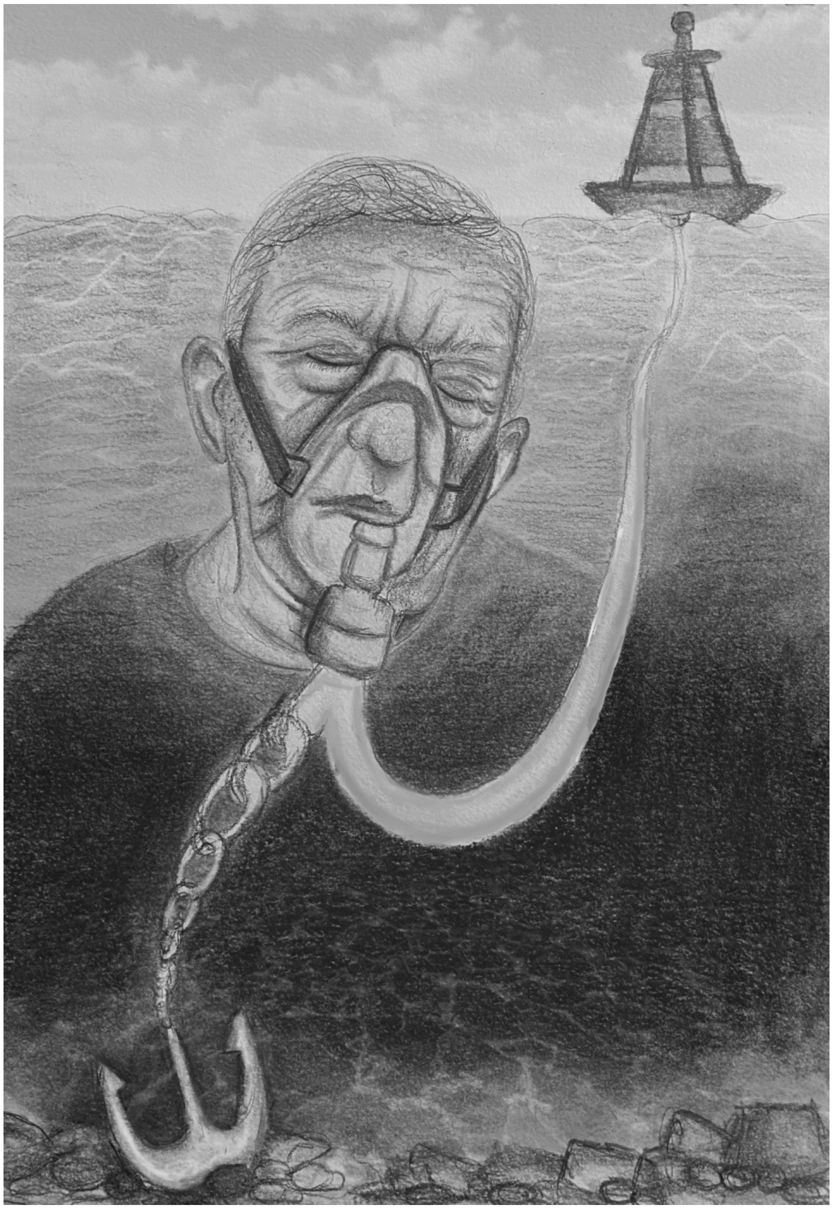

186x270mm (300 x 300 DPI)

Reporting checklist for qualitative study.

Based on the SRQR guidelines.

Instructions to authors

Complete this checklist by entering the page numbers from your manuscript where readers will find each of the items listed below.

Your article may not currently address all the items on the checklist. Please modify your text to include the missing information. If you are certain that an item does not apply, please write "n/a" and provide a short explanation.

Upload your completed checklist as an extra file when you submit to a journal.

In your methods section, say that you used the SRQRreporting guidelines, and cite them as:

O'Brien BC, Harris IB, Beckman TJ, Reed DA, Cook DA. Standards for reporting qualitative research: a synthesis of recommendations. Acad Med. 2014;89(9):1245-1251.

		Reporting Item	Page Number
**Title**			
	#1	Concise description of the nature and topic of the study identifying the study as qualitative or indicating the approach (e.g. ethnography, grounded theory) or data collection methods (e.g. interview, focus group) is recommended	Title page
**Abstract**			
	#2	Summary of the key elements of the study using the abstract format of the intended publication; typically includes background, purpose, methods, results and conclusions	1
**Introduction**			
Problem formulation	#3	Description and significance of the problem / phenomenon studied: review of relevant theory and empirical work; problem statement	2

For peer review only

Knutsen Glette M, et al. BMJ Open 2019; 0:e031297. doi: 10.1136/bmjopen-2019-031297

Purpose or research question	#4	Purpose of the study and specific objectives or questions	1-2
**Methods**			
Qualitative approach and research paradigm	#5	Qualitative approach (e.g. ethnography, grounded theory, case study, phenomenolgy, narrative research) and guiding theory if appropriate; identifying the research paradigm (e.g. postpositivist, constructivist / interpretivist) is also recommended; rationale. The rationale should briefly discuss the justification for choosing that theory, approach, method or technique rather than other options available; the assumptions and limitations implicit in those choices and how those choices influence study conclusions and transferability. As appropriate the rationale for several items might be discussed together.	2
Researcher characteristics and reflexivity	#6	Researchers' characteristics that may influence the research, including personal attributes, qualifications / experience, relationship with participants, assumptions and / or presuppositions; potential or actual interaction between researchers' characteristics and the research questions, approach, methods, results and / or transferability	10
Context	#7	Setting / site and salient contextual factors; rationale	2
Sampling strategy	#8	How and why research participants, documents, or events were selected; criteria for deciding when no further sampling was necessary (e.g. sampling saturation); rationale	2
Ethical issues pertaining to human subjects	#9	Documentation of approval by an appropriate ethics review board and participant consent, or explanation for lack thereof; other confidentiality and data security issues	3
Data collection methods	#10	Types of data collected; details of data collection procedures including (as appropriate) start and stop dates of data collection and analysis, iterative	2

For peer review only

Knutsen Glette M, *et al. BMJ Open* 2019; 0:e031297. doi: 10.1136/bmjopen-2019-031297

		process, triangulation of sources / methods, and modification of procedures in response to evolving study findings; rationale	
Data collection instruments and technologies	#11	Description of instruments (e.g. interview guides, questionnaires) and devices (e.g. audio recorders) used for data collection; if / how the instruments(s) changed over the course of the study	2
Units of study	#12	Number and relevant characteristics of participants, documents, or events included in the study; level of participation (could be reported in results)	2, Appendix A
Data processing	#13	Methods for processing data prior to and during analysis, including transcription, data entry, data management and security, verification of data integrity, data coding, and anonymisation / deidentification of excerpts	2
Data analysis	#14	Process by which inferences, themes, etc. were identified and developed, including the researchers involved in data analysis; usually references a specific paradigm or approach; rationale	2, 10
Techniques to enhance trustworthiness	#15	Techniques to enhance trustworthiness and credibility of data analysis (e.g. member checking, audit trail, triangulation); rationale	2,10
**Results/findings**			
Syntheses and interpretation	#16	Main findings (e.g. interpretations, inferences, and themes); might include development of a theory or model, or integration with prior research or theory	3-10
Links to empirical data	#17	Evidence (e.g. quotes, field notes, text excerpts, photographs) to substantiate analytic findings	3-8
**Discussion**			
Intergration with prior work, implications, transferability and contribution(s) to the field	#18	Short summary of main findings; explanation of how findings and conclusions connect to, support, elaborate on, or challenge conclusions of earlier scholarship; discussion of scope of application /	8-10

For peer review only - http://bmjopen.bmj.com/site/about/guidelines.xhtml

		generalizability; identification of unique contributions(s) to scholarship in a discipline or field	
Limitations	#19	Trustworthiness and limitations of findings	10
**Other**			
Conflicts of interest	#20	Potential sources of influence of perceived influence on study conduct and conclusions; how these were managed	11
Funding	#21	Sources of funding and other support; role of funders in data collection, interpretation and reporting	11

The SRQR checklist is distributed with permission of Wolters Kluwer © 2014 by the Association of American Medical Colleges. This checklist was completed on 26. April 2019 using https://www.goodreports.org/, a tool made by the EQUATOR Network in collaboration with Penelope.ai

For peer review only

Knutsen Glette M, *et al. BMJ Open* 2019; 0:e031297. doi: 10.1136/bmjopen-2019-031297

BMJ Open

The Impact on Staff of Providing Non-Invasive Advanced Respiratory Support During the Covid-19 Pandemic– A Qualitative Study in an Acute Hospital

Journal:	*BMJ Open*
Manuscript ID	bmjopen-2021-060674.R1
Article Type:	Original research
Date Submitted by the Author:	10-Apr-2022
Complete List of Authors:	Wenzel, David; University of Leicester<br>Bleazard, Lucy; University of London<br>Wilson, Eleanor; University of Nottingham, Faculty of Medicine & Health Sciences<br>Faull, Christina; LOROS Hospice
**Primary Subject Heading**:	Palliative care
Secondary Subject Heading:	Respiratory medicine
Keywords:	COVID-19, QUALITATIVE RESEARCH, Public health < INFECTIOUS DISEASES

SCHOLARONE™
Manuscripts

I, the Submitting Author has the right to grant and does grant on behalf of all authors of the Work (as defined in the below author licence), an exclusive licence and/or a non-exclusive licence for contributions from authors who are: i) UK Crown employees; ii) where BMJ has agreed a CC-BY licence shall apply, and/or iii) in accordance with the terms applicable for US Federal Government officers or employees acting as part of their official duties; on a worldwide, perpetual, irrevocable, royalty-free basis to BMJ Publishing Group Ltd ("BMJ") its licensees and where the relevant Journal is co-owned by BMJ to the co-owners of the Journal, to publish the Work in this journal and any other BMJ products and to exploit all rights, as set out in our licence.

The Submitting Author accepts and understands that any supply made under these terms is made by BMJ to the Submitting Author unless you are acting as an employee on behalf of your employer or a postgraduate student of an affiliated institution which is paying any applicable article publishing charge ("APC") for Open Access articles. Where the Submitting Author wishes to make the Work available on an Open Access basis (and intends to pay the relevant APC), the terms of reuse of such Open Access shall be governed by a Creative Commons licence – details of these licences and which Creative Commons licence will apply to this Work are set out in our licence referred to above.

Other than as permitted in any relevant BMJ Author's Self Archiving Policies, I confirm this Work has not been accepted for publication elsewhere, is not being considered for publication elsewhere and does not duplicate material already published. I confirm all authors consent to publication of this Work and authorise the granting of this licence.

For peer review only

The Impact on Staff of Providing Non-Invasive Advanced Respiratory Support During the Covid-19 Pandemic– A Qualitative Study in an Acute Hospital

For peer review only

Correspondence to: Dr David Wenzel, LOROS Hospice, Groby Road, Leicester, LE3 9QE
David.wenzel@nhs.net

Dr Lucy Bleazard, School of Medicine, University of Leicester, Leicester, UK

Dr Eleanor Wilson, School of Health Sciences, University of Nottingham, Nottingham, UK

Professor Christina Faull, Palliative Care, LOROS Hospice, Leicester, UK

Main Text Word Count: 4299

Objectives: To explore the experiences of healthcare workers of providing Non-Invasive Advanced Respiratory Support (NARS) to critically unwell patients with covid-19.

Design: A qualitative study drawing on a social constructionist perspective using thematic analysis of semi-structured interviews.

Setting: A single acute UK NHS Trust across two hospital sites.

Participants: Multidisciplinary team members in acute, respiratory and palliative medicine.

Results: 21 nurses, doctors (juniors and consultants) and physiotherapists described the provision of NARS to critically unwell covid-19 patients as extremely challenging. The main themes were of feeling ill prepared and unsupported, a need to balance complex moral actions, a sense of duty to patients and their families. The impact on staff was profound and findings are discussed via a lens of moral injury. Injurious events included staff feeling they had acted in a way that caused harm, failed to prevent harm or had been let down by seniors or the Trust. Participants identified factors that mitigated adverse impact.

Conclusions: Although many of the issues described by participants are likely immutable components of healthcare in a pandemic there were several important protective factors that emerged from the data. Experience, debriefing and breaks from covid-19 wards were valuable to participants and successfully achieving a peaceful death for the patient was often viewed as compensation for a difficult journey. These protective factors may provide modelling for future education and support services to help prevent moral injury – or aide in its recovery.

Registration: registered on the Open Science Framework 10.17605/OSF.IO/XB8YH

Strengths and Limitations

- Single Centre Study
- The lead author had pre-existing relationships with study participants
- The lead author, under strict supervision, practiced a reflexivity based approach to data collection and analysis with objectivity benchmarking from the second author.

For peer review only

INTRODUCTION

Non-invasive advanced respiratory support (NARS), including continuous positive airway pressure (CPAP) or bilevel positive airway pressure (BiPAP, sometimes referred to as simply non-invasive ventilation (NIV)) is an essential component of modern clinical practice[1]. It is used for the treatment of critically ill patients with a range of common conditions including chronic obstructive pulmonary disease (COPD), pulmonary oedema and, more recently, covid-19[2,3].

NARS is used extensively in the treatment of respiratory failure due to covid-19. It is a first-line treatment for those (largely younger people) who would be suitable for escalation to intubation should this be required and for those (largely older people) for whom it constitutes the ceiling of treatment[3]. The mortality rate is around 75% amongst those not considered appropriate for invasive ventilation[4] and the treatment carries significant burden[5]. A considerable number of these patients die either still using NARS or after its withdrawal; a procedure which represents a time of great uncertainty for both staff and patients[6].

Despite this, there is little data or guidance for clinicians caring for these patients at the end of life – perhaps because of the lack of research into symptom control in this area of care[7,8].

Quantitative studies of the impact of covid-19 on healthcare workers have shown high levels of post-traumatic stress symptoms[9], notably greater than in other infectious disease outbreaks[10]. This study looks to explore the causes of this impact in the highest mortality area of covid-19 care – critically ill patients requiring NARS who were not suitable for escalation to invasive intubation[4]. There is a paucity of data both on how best to provide care for these patients as they die and what impact this has on those who care for them.

METHODS

Design

A qualitative design drawing on a social constructionist perspective using semi-structured interviews[11,12]. A social constructivist perspective was used to reflect the role that participants had in forming their own truth. Whilst each individual's truth and the interpretation presented is valid, it may lack a universality when applied to other social contexts[13]. Initially participants described a memorable patient who had used NARS and died and an iteratively developed topic guide facilitated further exploration of issues, practices and experiences[12]. This article was evaluated before publication against the Standards for Reporting Qualitative Research (SRQR) reporting guidelines[14].

Settings and Participants

Doctors, nurses (from ward nursing and specialist palliative care nursing) and physiotherapists from acute medicine, respiratory medicine and palliative care were recruited using a purposive sampling approach from wards involved in the delivery of NARS in two hospital sites of an acute national health service (NHS) Trust in England. See appendix A for full details of participants. In order to enable the broadest diversity of participation of staff groups participants were invited to take part individually or in pairs or groups if preferred. A maximum of 37 participants were allowed for in the study design

For peer review only - http://bmjopen.bmj.com/site/about/guidelines.xhtml

with premature closure to recruitment to occur on thematic saturation i.e. that no further new codes were added to subsequent transcripts at the analysis stage. This saturation end point prevents unnecessary data collection that does not add to the richness of the themes[15].

Participants had a range of experience with NARS. Four participants had less than five years' experience of working with NARS and 17 had more than five years' experience. Two of the consultant participants had worked in the founding group for introduction of NARS services to the acute hospital. Participants from the acute medicine hospital site worked within a medical unit dedicated to NARS care for the pandemic. Participants from the respiratory hospital worked either on the admissions unit or dedicated covid-19 NARS wards.

Patient and Public Involvement

No patient or public involvement was sought in the creation of this study as it focused solely on the impact of healthcare workers and contained no patient or public participation in data collection. Results from this study will be disseminated to participants via internal email.

Data Collection

19 interviews of 21 participants were undertaken by telephone (n=2), Microsoft Teams (n=16) or face to face interview (n=1) audio recorded (encrypted voice recorder, Olympus DS-3500), and transcribed verbatim. One group interview with three participants was performed. A summative version of the topic guide is included in appendix B.

Data Analysis

Thematic development was contemporaneous but dynamic and in parallel with data collection based on a principles of constant comparison[16], supported by qualitative coding software, MAXQDA Plus 2020 (V20.4.0). Inductive coding and development of the initial coding frame was undertaken by DW and LB with iterative group (including CF and EW) discussion. The whole team sought to identify areas of agreement and commonality between participants whilst recognising the range of perspectives and experiences described. These, sometimes even contradictory views, were crystalised into our final data analysis and added a profound richness to the data that was facilitated by the semi-structured interview format[17]. Data collection was completed when thematic saturation was achieved.

Ethics

NHS Research Ethics Committee review was not required as the study involved only NHS staff. However, local sponsorship was provided by the University Hospitals of Leicester (EDGE ID 138908). All participants provided written consent at the start of, or prior to, their interviews.

RESULTS

For peer review only - http://bmjopen.bmj.com/site/about/guidelines.xhtml

For peer review only

In all interviews participants discussed both the high level and the harmful consequences of the emotional, physical and psychological impact levied upon them and their colleagues by the covid-19 pandemic.

> *"… you know when you wear the FFP3[1] masks? I can smell that smell [and] it takes me back to the first lady that I'd been involved in a CPAP withdrawal for and I just remember it…" (Specialist Palliative Care Nurse)*

> *"… it massively impacted me emotionally… I got really anxious. I wasn't really sleeping. I wasn't eating much. I had a bit of talking therapy" (Junior Doctor)*

> *"I was emotionally broken. I remember crying at the end of that weekend. I was, I was like 'Oh my God' and I had to, I had to actually reach out to my consultant colleagues to say, 'I feel broken' and they were like, 'that's normal'." (Acute Medicine Consultant)*

Five themes emerged from the data that describe the causes of such impact and factors that may protect against harmful consequences. The group interview did not yield different codes or themes to the individual interviews and therefore will not be further identified in the results.

Present throughout the data was the concept of repetition, the moral harms and impact of this work affected staff multiple times a day. This did not explain how the work was injurious and so was not incorporated into a theme, but rather underlined the impact of all the themes.

> *"I remember when cases started coming back again recently … and immediately everyone felt that pit in their stomach… 'here we go again' and what does that mean for us? It ultimately means one thing, they're all going to die. For us, it's not necessarily a death it's the amount of death, the amount of withdrawals, the amount of things that we should see in maybe thirty years of nursing - we've had in a very compressed space of time. So, it makes you feel ill. (Palliative Care Nurse)*

Ill Prepared and Unsupported

Participants expressed concern over educational needs that were not met prior to deployment. Whilst it was noted that a lack of education around end-of-life care and NARS, including ethics and clinical reasoning, predated the pandemic, the effects of this were compounded by covid-19. Participants relied on informal teaching from colleagues and other sources (such as popular literature) to make up for this shortfall.

> *"Actually, I learn most of what I know about death and dying from working with my palliative care colleagues" (Physiotherapist)*

There was a perceived lack of opportunity for debriefing with most reflection after traumatic events being informal between colleagues. There was often a feeling that more

[1] FFP3 – Filtering Face Pieces 3, a tight-fitting filtration mask used as part of personal protective equipment during exposure to aerosol generating procedures.

For peer review only - http://bmjopen.bmj.com/site/about/guidelines.xhtml

could be done at an organisational level to support staff in areas with high levels of NARS covid-19 care.

> *"I'm not sure we got a lot from the Trust... I think there was very little formal support. I think that compared to some areas we did very badly on getting debriefs and things organized." (Acute Medicine Consultant)*

Being short of staff and poor continuity of care were referenced by many participants as having negative impacts on their ability to provide care.

Moral Actions

Participants spoke often of actions undertaken during the pandemic that had a moral underpinning; acts that balance burden and benefit for the patient, acts that they were compelled or coerced to perform; and acts they were unable to complete.

NARS was a treatment regarded as unlikely to be successful with significant treatment burden and was often felt to be started due to a lack of other options, rather than as an act in keeping with the patient's best interests. Some participants reflected that starting NARS was a way for staff to cope personally with so many deaths,

> *"You've forced a treatment that is unpleasant on somebody... And sometimes it feels like you're assaulting them... [because] people just desperately don't want another person to die. But sometimes there's just the feeling of helplessness - I think that they're feeling that [starting CPAP] is a way that they can manage that feeling of the tsunami of dying." (Palliative care consultant)*

The decision to continue NARS weighed on participants in a similar fashion and even decisions to change interface made interviewees feel complicit in this burdensome treatment.

> *"The mask wasn't giving her much symptom relief, it wasn't giving her much support with oxygenation. So we went for the hood ... to see if it made a difference A) to her symptoms and B) to her clinical condition ... It didn't make a difference." (Physiotherapist)*

Over half of participants reported concerns they were giving patients and their families false hope, because the chance of recovering was minimal.

> *"Very often it's just the fact that the family are holding onto that chink of hope that something is dramatically going to turn around and the patient will improve. And you know it's not going to happen 'cause you've been there many times before." (Respiratory consultant)*

Communicating the poor prognosis and the imminence of death weighed on participants' minds:

> *"... you're looking someone in their eyes and telling them that they're dying. So that's the difficulty." (Respiratory Consultant)*

If CPAP treatment became too burdensome, with no chance of recovery, it was often withdrawn on the advice of senior clinicians or at the request of the patient themselves. The

For peer review only - http://bmjopen.bmj.com/site/about/guidelines.xhtml

process of withdrawal was often described as traumatising to staff. Participants felt a responsibility to withdraw the mask and enable the patient to die as peacefully and symptom free as possible but anticipating a patient's needs and to manage any distress was challenging.

Healthcare workers had a range of feelings after withdrawals. Some reported an immense sadness. The removal of the mask emphasised the teams' perceived failure to save that person's life. Some saw the withdrawal as an opportunity to create peace and a natural death, regarding it as essential for holistic palliative care. A minority of participants reported feeling responsible for causing the death by removing the mask - but most participants discussed knowing that others felt this way, even if they themselves did not.

> *"... being that person who takes it off, it can sometimes feel like you're the person ending that patient's life" (Physiotherapist)*

The visiting restrictions participants described varied throughout the pandemic. At times only patients dying *and* not on NARS were allowed end of life visitation. The recognition of death was often felt to be delayed and late in the dying process which further complicated visiting. Participants spoke extensively about restricting visitation from relatives during the patients' admission and especially when they were dying. This had a profound impact on some.

> *"But just to keep a family member away from their dying relative is probably one of the hardest things I've ever had to do." (Acute Medicine Consultant)*

Loss of Professional Autonomy

Autonomy was often felt to have been lost at the expense of managing the patients' families' expectations and readiness for the death of their loved one. It often became clear to clinicians and their teams that death was inevitable (based on oxygen requirements, ventilator dependency, work of breathing and clinical experience) before the family were ready to accept it. For patients who deteriorated despite NARS its withdrawal was generally seen as the logical/ethical clinical decision due to its progressive loss of symptomatic relief and increasing treatment burden (claustrophobia, impact on communication, breathlessness, anxiety). There were also professional concerns around prolonging and medicalising the dying process. These approaches were sometimes in tension with family views about the value of continuing NARS:

> *"I think the family were more so reluctant themselves to have the CPAP mask removed. So for them I guess it provided them a sense of comfort that at least everything was done till the very end." (Ward Nurse)*

A huge amount of communication was dedicated to ensuring that families understood their loved one's condition and were enabled to accept the clinical recommendations. If families could not concur or be helped to be ready prior to their loved one's death an extreme amount of distress was reported.

> *"... they wanted his wife to be able to see him over Facetime and they had the phone with the wife, in his face, while he was dying and gasping... they had removed the*

For peer review only - http://bmjopen.bmj.com/site/about/guidelines.xhtml

CPAP…. I can remember this distinct memory of wailing and screaming and I'm just sitting there trying to make it stop and the patient died, he died, whilst the wife was in [his] face because they wanted to see his face…" (Specialist Palliative Care Nurse)

Even when families were accepting of the clinical decisions being made, staff often felt their range of options were limited due to inheriting decisions from the previous shifts or earlier in the chain of care.

"In my own mind I would think, 'why have they been put on CPAP?'. …But there wasn't somebody I could have a disagreement with because the decision had been made 2, 3, 5 days earlier." (Acute Medicine Consultant)

Whilst respecting senior decision-makers and the complexity of their role, there were many references to staff being involved in clinical decisions they would not make themselves and the impact that this had on them.

"I'm probably more distressed by the people who I felt I supported a decision, or was coerced it felt like, into helping them have CPAP when I thought they were dying in the next few hours." (Palliative Care Consultant)

This was often due to the uncertainty of the clinical situation and at times led to conflict within the multi-disciplinary team (MDT) about the appropriateness of continuing therapy and prolonging or medicalising death. Disagreements were often reported as not openly discussed and the data suggest that this resulted in little awareness from senior decision makers.

"It's quite nice getting their opinions but I wouldn't necessarily do what they wanted if it wasn't what I wanted to do." (Respiratory Consultant)

"I know from a nursing perspective a lot of the nurses felt uncomfortable… I feel like a medical decision is probably very different to how the nurses felt." (Ward Nurse)

Participants frequently described their difficulty in controlling the severe symptoms that were caused by the patients' illness. Their efforts were further complicated by the impact of personal protective equipment (PPE) requirements.

"And became massively tachypnoeic and was trying to sit up at that point to get his breath, which was horrific. So, and the difficulty being…you've got to put on [PPE] to get in there." (Specialist Palliative Care Nurse)

These symptoms often occurred in patients with little history of ill health and in the context of rapid clinical decline. This made them more poignant to the staff that witnessed them.

Duty

Exposure to pandemic experiences was often viewed in the context of a sense of duty, to patients and their families. The moral actions participants undertook were viewed in the context of a deep sense of dedication to doing the 'right' thing and a responsibility for how families would perceive the death of their loved ones

For peer review only

For peer review only - http://bmjopen.bmj.com/site/about/guidelines.xhtml

"I think it's only human to try and avoid the difficult decisions but I think it's important that we have a duty of care where we try and address things that are difficult." (Junior Doctor)

"… you're guiding this experience for someone. And how are they going to look back on it? And are they going to look back on it and go, 'Yeah, that was OK.' Or are they going to look back and say, 'that was a disaster'." (Palliative Care Consultant)

This sense of duty led to actions by staff significantly beyond their usual role; acting as substitute family; partaking in end-of-life religious rituals; sitting in full PPE while a patient died; or taking part in end-of-life conversations that healthcare workers are rarely party to:

"… you effectively become, the nursing staff and the medical team, effectively become the surrogate family" (Junior Doctor)

"But prior to covid you wouldn't sit and listen to somebody else tell their Mum all their things that they want to in the minutes before she dies… you wouldn't witness all of that." (Palliative Care Consultant)

Ultimately, it appeared this sense of duty was often the driving factor for perseverance from otherwise expended staff:

"It was very much a 'we've just got to get on with this, we've just gotta get through this as best we can, remember that all of these patients are relying on us to make them comfortable'." (Palliative Care Nurse)

Protective Factors

Participants discussed successes they had during the pandemic and the factors that facilitated them. These seemingly buoyed their perceptions of their teams' actions and led to greater satisfaction with care provision, providing some protection from the ongoing strains of NARS care in the pandemic.

Dying peacefully was often referenced as a compensation for the overall nature of a patient's clinical journey. Ensuring minimal symptoms, family presence and every avenue of care had been explored gave participants a sense of accomplishment. The earlier clinical decisions were made, and the more clearly they were communicated, the more associated they were with better patient care:

"I had a case on [a ward] who went on and did a night on CPAP and didn't tolerate it and they stopped it overnight at two o'clock in the morning. And actually, he then went home and died at home [which was his wish]. *But my point more is that they sort of clocked... They didn't persevere. They clocked early that this wasn't going well. They got out." (Palliative Care Consultant)*

Higher levels of clinical experience were associated with increased resilience and surety around clinical decision making.

"It's a decision that you make. I can't predict the future anymore than anybody else can. But it's a case of you have to make a decision. And that's part and parcel of

For peer review only - http://bmjopen.bmj.com/site/about/guidelines.xhtml

having been a consultant and having the luxury of experience." (Acute Medicine Consultant)

Regardless of whether death was peaceful or not participants spoke highly of their colleagues' support afterwards. This support was often informal, based on local team dynamics and often individuals, but felt to be a valuable part of the recovery process:

"We often talk to [name]. She is really good at asking people how they are. People like talking to her, she's very sympathetic. And then I know the matron is very much on hand to talk to the nurses" (Respiratory Consultant)

"Some of the senior nurses were just fabulous, fabulous people, and we were being massively supported by them as well, as much as they would say we supported them. There were days when I was on my knees going "I just can't come back to this," you know." (Specialist Palliative Care Nurse)

Some participants discussed the benefits they had received from respite away from the high acuity environment of the wards. All participants who referenced this were doctors, most consultants with other clinical or academic commitments and had concerns for others in the team who did not have this.

"It doesn't seem to have a particular effect on me but maybe I just had… less involvement over the last six months and that's allowed me to sort of lick my wounds and feel better for it." (Acute Medicine Consultant)

Some participants reported the use of practices to protect themselves or others from the impact of their work. Participants reported self-identifying that they were unable to complete certain tasks (mask withdrawals and breaking bad news especially) and either delaying or delegating the task and senior decision makers also reported identifying staff who needed tasks reallocating.

"That's not always possible, but we also try to recognize that in our team… when we've done several difficult ones in a day, or in a few days, that actually you may not be the right person to do the next one a few hours later. Because you might not have enough of yourself to give." (Palliative Care Consultant)

DISCUSSION

Our data demonstrated significant personal impact on participants in keeping with quantitative studies[18] but has added to this literature by identifying *how* working in the pandemic had this effect on staff supporting people with respiratory failure outside of intensive care units. Participants were forced by circumstance to engage in complex moral actions balancing burden and benefit while treating patients with severe symptoms. This impact was compounded by the overwhelming volume of patients and the duty staff felt to help them and their families. This complex care environment often resulted in a perceived loss of professional autonomy – further confounding participants' moral actions.

Analysis revealed significant congruence between our data and the existing canon of knowledge around the theory of moral injury. Moral injury refers to the impact felt when a

For peer review only - http://bmjopen.bmj.com/site/about/guidelines.xhtml

person experiences events not in keeping with their own moral code, or conscience. The concept of moral injury has become closely linked to the pandemic with statements of concern about the prevalence of moral injury from governments and professional bodies[19,20]. These events are often subdivided into acts of commission, omission and betrayal[21]. Many of the actions undertaken by participants were moral actions that balanced cost or burden and benefit: restricting visiting, initiating high burden treatment and withdrawing treatment.

Being forced to balance a patient's desire and right to see their family before they die and the safety of their family and society may result in a morally 'correct' action, but the process of enacting this and the witnessed distress it causes is injurious to the practitioner. This is consistent with an act of commission, an action a person is compelled to undertake by seniors or the environment of care[22].

The value of NARS was a balance of burdensome treatment and of hope of possible recovery and symptom relief. An inability to stop death, to control symptoms adequately or act in the best interest of the patient weighed heavily on participants. These represent acts of omission, acts that are not undertaken by healthcare workers due to the environment in which they are working, and this led participants to feel as if their care was lacking. It should be noted that there is currently no literature to support the role of NARS as effective symptom relief in covid-19 and only limited evidence in COPD[8].

Some of the most challenging experiences participants described were around the loss of autonomous decision making. Feeling coerced into forcing treatment on a patient by colleagues or feeling unable to withdraw due to family readiness impacted participants who then felt complicit in the suffering that ensued. This loss of autonomy represents both an act of omission and an act of commission.

Participants expressed concern over educational needs that had not been met prior to deployment to covid-19 wards as well as a lack of formal debriefing opportunity after challenging cases. This represents an act of betrayal, where a trusted other creates exposure to injurious events – often through lack of preparation or support[23]. However, in contrast with existing literature[24], no references were made by participants to personal protective equipment availability and only one participant described concerns over level of exposure and the risk of catching covid-19. Betrayal as an origin of moral injury was less prevalent across interviews than reflections on commission and omission.

Literature around moral injury describes those affected as 'witnesses'[22] to suffering. Our data suggested that the impact from injurious events was facilitated by the participants sense of ownership over the traumas they witnessed. The duty that they felt to provide good care and support to families was key in the mechanism of their suffering. This sense of 'ownership' should be considered in future works examining moral injury in healthcare workers.

Present throughout our data was the concept of repeated injury. It was not only that morally injurious events occurred, but that they occurred much more frequently than in usual circumstances. This was a significant finding present in every interview that appeared to exacerbate the impact felt by healthcare workers during the pandemic.

Moral Injury represents a wide spectrum of impact, from self-limiting to chronically debilitating and requiring professional treatment[25]. This spectrum was present in our data. Some participants were unaffected, some had persistent symptoms and a minority spoke about requiring formal treatment to recover (psychological therapy).

There was great openness from participants to discuss the impact that providing end of

For peer review only - http://bmjopen.bmj.com/site/about/guidelines.xhtml

life care in a pandemic had on them. This in and of itself may be indicative of the lack of formal structures in place to help staff recover. Lack of support in recovery was described frequently but there was no clear consensus over what would be most helpful in this regard. The impact on staff of low impact/high burden treatments is rarely considered in the literature but may need to form a key part of any support packages used in the future. The low level efficacy of NARS was anecdotally apparent to staff but not apparent in formal evidence throughout much of the pandemic and only recent papers formally question its use in 'do not intubate' patients[3,4,5].

Our findings identify several issues that may form key components of preventative strategy including: education around NARS use and withdrawal[26], the ethical reasoning around NARS and how we prepare staff for patient death. Perceived poor staff continuity and inadequate staffing levels exacerbate issues within this context but may not be easily addressed.

A great many of the components of moral injury to staff were, and remain, seemingly unavoidable. As the patients suffered, so did their caregivers. This may be a component of pandemic healthcare[27,28]. However, how we prepare, support and aide recovery warrants further investigation. Participants described several coping mechanisms, debriefing requirements and post injury recovery techniques that could aide with workforce planning moving forward.

A peaceful and holistically provided for patient death was often viewed by participants as compensation for a tumultuous journey. The role NARS may have in a peaceful death as symptom control for breathlessness is an understudied phenomenon[29,30,31]. Further research on the role of NARS in end-of-life care will be important to continue to improve patient care and support our staff in facilitating good end-of-life care.

LIMITATIONS

The principle investigator, DW, is a doctor with extensive experience working in the NARS covid-19 wards with existing relationships with the participants. While this was a considerable advantage for recruitment and openness between participant and researcher, we recognise that this could have influenced the data gathered. CF is primarily an academic palliative care physician who spent no time in the acute trust during the pandemic. Under CF's supervision DW practiced a reflexivity-based approach to limit the impact of personal bias on data collection and analysis – this included keeping a reflexivity journal, regular supervision meetings and splitting data collection and analysis with a second researcher, LB. LB had no pre-existing relationships with the research participants and limited covid-19 exposure. LB carried out five of the interviews and analysed three independently. LB and DW jointly coded five of the transcripts and thematic generation took place in team meetings. Data was collected from only one hospital trust.

A degree of recall bias may have influenced data collection, especially as participants were asked to recall a memorable patient. However, this methodology allows significant in depth exploration if scenarios that are impactful to the participant, which was the aim of the research.

CONCLUSION

This study identified the considerable impacts on health care staff caring for patients who

For peer review only - http://bmjopen.bmj.com/site/about/guidelines.xhtml

For peer review only

required NARS and died due to covid-19. It has identified the multifactorial origins of distress and harm to staff and explores this within the context of the duty that staff felt towards their patients which augments the current understanding of moral injury.

A great many of the factors that impacted on staff are immutable and unavoidable components of severe respiratory disease. Being unable to reverse an illness process, witnessing distress and breaking bad news are regrettable essentials of this work. However, the role of staff as active agents of distress – restricting visitation, starting high burden treatments, witnessing family distress and a sense of involvement in prolonging suffering or causing death may require further reflection should the pandemic worsen, or a new one occurs. Importantly the study was able to identify several protective factors that may prevent, reduce and aide recovery from injurious events. Understanding how to protect and minimise psychological injury to our workforce in these impactful circumstances will be essential in safeguarding the future of healthcare provision.

For peer review only

For peer review only - http://bmjopen.bmj.com/site/about/guidelines.xhtml

Transparency Declaration
The lead author, Dr David Wenzel, affirms that this manuscript is an honest, accurate, and transparent account of the study being reported; that no important aspects of the study have been omitted; and that any discrepancies from the study as planned (and, if relevant, registered) have been explained.

Contributorship Statement
Contributors: DW and CF conceptualised and developed the project. The protocol was authored by DW, who is the guarantor, and reviewed/edited by CF. Data collection tools were designed by all authors. Data collection was performed principally by DW supported by LB. Database creation was principally by DW, supported by LB. Data analysis was performed principally by DW with support from LB, EW and CF (contribution level in order). Thematic analysis was performed equally by DW and LB with further contribution from CF and EW. The primary report was authored by DW with editing and review by EW, CF and LB (contribution level in order).

Competing Interests
We have read and understood the BMJ policy on declaration of interests and declare the following interests: None.

Funding
The lead author's current posting, academic clinical fellow, is a funded role by the National Institute of Health Research (NIHR). The NIHR had no input in study creation, design or reporting. Funding award reference ACF-2019-11-007.

Data Sharing Statement
Data are available upon reasonable request. All raw materials including de-identified raw data, coding and thematic analysis can be obtained by contacting lead author Dr David Wenzel via email at.

References
1 Pierson D. History and Epidemiology of Non-invasive Ventilation in an Acute Care Setting, *Respir Care*, 2009;01;54(1):40-52.

2 National Institute for Health and Care Excellent (NICE), 2019, Chronic obstructive pulmonary disease—Management of chronic obstructive pulmonary disease in adults in primary and secondary care, NICE guideline NG115, https://www.nice.org.uk/guidance/NG115

3 Mahase E. Covid-19: CPAP reduces need for invasive mechanical ventilation in patients requiring oxygen, study finds BMJ, 2021;374:n1950

4 Vaschetto R, Barone-Adesi F, Racca F, et al. Outcomes of COVID-19 patients treated with continuous positive airway pressure outside the intensive care unit, *ERJ Open Research,* 2021,07:00541-2020

For peer review only - http://bmjopen.bmj.com/site/about/guidelines.xhtml

5 Bradley P, Wilson J, Taylor R, et al. Conventional oxygen therapy versus CPAP as a ceiling of care in ward-based patients with COVID-19: a multi-centre cohort evaluation, *EClinicalMedicine,* 2021;10:40;101122

6 Hough A, Dell GD, Blaber M, et al. Beyond the mask: a multidisciplinary reflection on palliating patients with COVID-19 receiving continuous positive airway pressure ventilation, *Int J Palliat Nurs,* 2020;10:2;26(7):336-340

7 Smith TA, Davidson PM, Lam LT, et al. The use of non-invasive ventilation for the relief of dyspnoea in exacerbations of chronic obstructive pulmonary disease; a systematic review. Respirology. 2012 Feb;17(2):300-7

8 Wenzel D, Bleazard L, Pepper C, et al. Symptom Management When Non-Invasive Advanced Respiratory Support is Used During End-of-Life Care: A Systematic Review. medRxiv 22273098 [Preprint] March 29 2022 [cited 2022 April 22] doi: https://doi.org/10.1101

9 Mosheva M, Gross R, Hertz-Palmor N, et al. The association between witnessing patient death and mental health outcomes in frontline COVID-19 healthcare workers. *Depress Anxiety* 2021;4:38(4):468-479.

10 Lamb D, Gnanapragasam S, Greenberg N, et al. Psychosocial impact of the COVID-19 pandemic on 4378 UK healthcare workers and ancillary staff: initial baseline data from a cohort study collected during the first wave of the pandemic. *Occupational and Environmental Medicine,* 2021;6:78:801-808.

11 Charmaz K. Constructing Grounded Theory; A Practical Guide Through Qualitative Analysis, London, Sage Publications, 2006: 125-131.

12 Rubin HJ, Rubin IS. Qualitative Interviewing: The art of hearing data, London, Sage Publications, 2005: 64-78

13 Jackson P. From function to use. In: Jackson P, Paul Jackson, Web 2.0 Knowledge Technologies and the Enterprise, Oxford UK, Chandos Publishing 2010:155-196

14 O'Brien BC, Harris IB, Beckman TJ, Reed DA, Cook DA. Standards for reporting qualitative research: a synthesis of recommendations. *Acad Med*. 2014;89(9):1245-1251

15 Saunders B, Sim J, Kingstone T, et al. Saturation in Qualitative Research: exploring its conceptualisation and operationalisation. *Quality and Quantity,* 2018;52;1893-1907

16 Charmaz K. Constructing Grounded Theory; A Practical Guide Through Qualitative Analysis, London, Sage Publications, 2006: 42-71.

17 Barbour R S. Checklists for improving rigour in qualitative research: a case of the tail wagging the dog? *BMJ,* 2001; 322 :1115

18 Hines SE, Chin KH, Levine AR, Wickwire EM. Initiation of a survey of healthcare worker distress and moral injury at the onset of the COVID-19 surge. *Am J Ind Med,* 2020;63(9):830-833

For peer review only

For peer review only - http://bmjopen.bmj.com/site/about/guidelines.xhtml

19 British Medical Association (BMA), 2021, Moral Distress and Moral Injury: recognising and tackling it for UK doctors. https://www.bma.org.uk/media/4209/bma-moral-distress-injury-survey-report-june-2021.pdf [Accessed April 2022]

20 Risk Communication and Behavioural Insights Sub-Group, (2021) Technical Advisory Group: moral injury in health care workers during the COVID-19 pandemic, Llywodraeth Cymru/Welsh Government, https://gov.wales/sites/default/files/pdf-versions/2021/8/2/1628000224/technical-advisory-group-moral-injury-health-care-workers-during-covid-19-pandemic.pdf [Accessed April 2022]

21 Ford, EW. Stress, Burnout, and Moral Injury: The State of the Healthcare Workforce, *J Healthc Manag*. 2019;5-6:64;3;125-127

22 Cartolovni A, Stolt M, Scott PA, et al. Moral injury in healthcare professionals: A scoping review and discussion. *Nurs Ethics*. 2021;1:28(5);590-602.

23 Shay J. Moral Injury. *Psychoanal Psychol.* 2014:31;2;182-191

24 French L, Hanna P, Huckle C. "If I die, they do not care": U.K. National Health Service staff experiences of betrayal-based moral injury during COVID-19. *Psychol Trauma*. [preprint] December 07, 2021 https://doi.org/10.1037/tra0001134

25 Williamson V, Murphy D, Phelps A, et al. Moral injury: the effect on mental health and implications for treatment. *Lancet Psychiatry*. 2021;3:8(6), 453–455.

26 Faull C, Phelps K, Regen E, et al. Withdrawal of Ventilation at the patient's request in MND: Doctors views on the ethical challenges involved. *BMJ Support Palliat Care*. 2014;4:A39

27 Bernstein M, Hawryluck L. Challenging beliefs and ethical concepts: the collateral damage of SARS. *Crit Care*. 2003;5:7;269

28 Leong IY, Lee AO. The challenge of providing holistic care in a viral epidemic: opportunities for palliative care. *Palliat Med*. 2004:18:12-18

29 Smith TA, Davidson PM, Jenkins CR, et al. Life behind the mask: the patient experience of NIV. *Lancet Respir Med* 2015;3:8–10.

30 Craig D, Steve B, Mark E, et al. BTS/ICS Guidelines for the Ventilatory Management of Acute Hypercapnic Respiratory Failure in Adults, *Thorax* 2016;4:71 Suppl 2

31 Smith TA, Agar M, Jenkins CR, et al. Experience of acute noninvasive ventilation—insights from 'Behind the Mask': a qualitative study, *BMJ Support Palliat Care*, 2019;9:e11.

For peer review only

For peer review only - http://bmjopen.bmj.com/site/about/guidelines.xhtml

Appendix A

Demographic Traits	Female	Male
Ward Nurses	1	0
Specialist Nurses (Palliative Care)	5	0
Junior Doctors (registrar or below)	3	1
Consultants (Acute/General Med)	1	2
Consultants (Respiratory)	2	1
Consultants (Palliative Medicine)	2	1
Physiotherapists	1	1
Total	15	6

For peer review only

Appendix B – Summative Topic Guide

Reconfirm Consent

Before we start can I please confirm you have had a chance to read through the participant information sheet? Do you have any questions from this?

I would like to remind you at this point that your participation, while appreciated, is voluntary and you can stop the interview at any point you want. As you know to help with our data collection I will be audio recording this interview. I will turn on the audio recorder now.

Are you still happy to consent to being a part of this study as per the items in your signed consent form?

For peer review only

Introduction

Thank you for agreeing to participate in this interview/focus group. We are interviewing you to better understand your experiences and any challenges of providing care to dying patients who are using, or have recently used non-invasive advanced respiratory support (such as CPAP or BiPaP/NIV). There are no right or wrong answers to any of my questions, I am interested in your own experiences and thoughts.

Contextualisation

Can you please describe your role in the hospital and outline what sort of experience you have caring for patients on CPAP or NIV?

I would like you to think back to a memorable patient of yours – who received CPAP or NIV but died. Most of my questions will be about this one case, but feel free to talk about other patients as we go through.

Please, tell me about them.

How was the decision to start CPAP/NIV discussed with the patient/family?
Prompt – if you weren't part of that conversation, what things would it be important for you to tell a patient under your care before starting CPAP?

How did they react to the mask therapy being started?
Prompt: did they need any medication to make the mask more tolerable?

Did the mask help them/their symptoms?
Prompt: if you weren't present when it was started, do masks usually help with these symptoms?

Did the patient talk to you at all about having the mask?

Did the patient talk to you at all about their illness and what was happening?

What made you choose this patient over any of the others that you've treated?

What was your role with the patient in their last days?

Decision Making

Was your patient recognised to be dying before they died?
If So: Why were they thought to be dying?
If Not: How would you normally know a patient was dying? Why did that not happen in this case do you think?

Was there a decision made to remove the patients mask?
If So: Why?
If Not: Why not?

How did you feel about this decision?

How did the team discuss this decision and the plan?
Prompt: How was it discussed with the patient and their loved ones?

Were there any disagreements over whether mask therapy should be stopped or continued?
If Yes – What were they? How were they resolved?

Mask removal

Who physically removed the mask?

How was the removal carried out?
Prompt: were any medications given before or after? Did the patient receive oxygen after?

Do you think it makes a difference to the patient if they die with the mask in place or removed before they die? Why?

Does it have any impact on their family?

How do you feel about it?

Symptom management

Do you recall What symptoms the patient had?

Did the patient have symptoms managed in anyway?

Communication and Mask Removal
If mask left in place: I would like now for you to think of a patient who's mask was removed before they died.

Who was involved in the decision to remove the mask?
If patient not included: why was patient not included?

In your experience, which type of professional is normally responsible for physically taking the mask off?

Do you think this affects them? Is this something you have ever discussed with your colleagues?

For peer review only

Is there any sort of protocol people follow when removing NIV/CPAP? Or are plans provided by the medical team?

Do you think it is helpful to have a 'plan' for removing an NIV/CPAP mask? Why?

What things should be part of that plan?

How do you feel about removing a mask in circumstances where a patient is dying on NARS? Have you ever had a colleague discuss this with you?

<u>Reflection</u>

Did your patient have a 'good death'
Prompts: What made it a good death?
What would have made it a good death? What might have been done better in retrospect?

Has this work had any effect on you as an individual?
Possible prompts: is the effect cumulative? IS there an opportunity for you to debrief? Would that be useful?

To round up, can you think of one key lesson you would want people to know about taking care of patients receiving CPAP or NIV?

For peer review only

Reporting checklist for qualitative study.

Based on the SRQR guidelines.

Instructions to authors

Complete this checklist by entering the page numbers from your manuscript where readers will find each of the items listed below.

Your article may not currently address all the items on the checklist. Please modify your text to include the missing information. If you are certain that an item does not apply, please write "n/a" and provide a short explanation.

Upload your completed checklist as an extra file when you submit to a journal.

In your methods section, say that you used the SRQRreporting guidelines, and cite them as:

O'Brien BC, Harris IB, Beckman TJ, Reed DA, Cook DA. Standards for reporting qualitative research: a synthesis of recommendations. Acad Med. 2014;89(9):1245-1251.

		Reporting Item	Page Number
**Title**			
	#1	Concise description of the nature and topic of the study identifying the study as qualitative or indicating the approach (e.g. ethnography, grounded theory) or data collection methods (e.g. interview, focus group) is recommended	Title page
**Abstract**			
	#2	Summary of the key elements of the study using the abstract format of the intended publication; typically includes background, purpose, methods, results and conclusions	1
**Introduction**			
Problem formulation	#3	Description and significance of the problem / phenomenon studied: review of relevant theory and empirical work; problem statement	2

For peer review only

Knutsen Glette M, et al. BMJ Open 2019; 0:e031297. doi: 10.1136/bmjopen-2019-031297

Purpose or research question	#4	Purpose of the study and specific objectives or questions	1-2
**Methods**			
Qualitative approach and research paradigm	#5	Qualitative approach (e.g. ethnography, grounded theory, case study, phenomenolgy, narrative research) and guiding theory if appropriate; identifying the research paradigm (e.g. postpositivist, constructivist / interpretivist) is also recommended; rationale. The rationale should briefly discuss the justification for choosing that theory, approach, method or technique rather than other options available; the assumptions and limitations implicit in those choices and how those choices influence study conclusions and transferability. As appropriate the rationale for several items might be discussed together.	2
Researcher characteristics and reflexivity	#6	Researchers' characteristics that may influence the research, including personal attributes, qualifications / experience, relationship with participants, assumptions and / or presuppositions; potential or actual interaction between researchers' characteristics and the research questions, approach, methods, results and / or transferability	10
Context	#7	Setting / site and salient contextual factors; rationale	2
Sampling strategy	#8	How and why research participants, documents, or events were selected; criteria for deciding when no further sampling was necessary (e.g. sampling saturation); rationale	2
Ethical issues pertaining to human subjects	#9	Documentation of approval by an appropriate ethics review board and participant consent, or explanation for lack thereof; other confidentiality and data security issues	3
Data collection methods	#10	Types of data collected; details of data collection procedures including (as appropriate) start and stop dates of data collection and analysis, iterative	2

For peer review only

Knutsen Glette M, *et al. BMJ Open* 2019; 0:e031297. doi: 10.1136/bmjopen-2019-031297

		process, triangulation of sources / methods, and modification of procedures in response to evolving study findings; rationale	
Data collection instruments and technologies	#11	Description of instruments (e.g. interview guides, questionnaires) and devices (e.g. audio recorders) used for data collection; if / how the instruments(s) changed over the course of the study	2
Units of study	#12	Number and relevant characteristics of participants, documents, or events included in the study; level of participation (could be reported in results)	2, Appendix A
Data processing	#13	Methods for processing data prior to and during analysis, including transcription, data entry, data management and security, verification of data integrity, data coding, and anonymisation / deidentification of excerpts	2
Data analysis	#14	Process by which inferences, themes, etc. were identified and developed, including the researchers involved in data analysis; usually references a specific paradigm or approach; rationale	2, 10
Techniques to enhance trustworthiness	#15	Techniques to enhance trustworthiness and credibility of data analysis (e.g. member checking, audit trail, triangulation); rationale	2,10
**Results/findings**			
Syntheses and interpretation	#16	Main findings (e.g. interpretations, inferences, and themes); might include development of a theory or model, or integration with prior research or theory	3-10
Links to empirical data	#17	Evidence (e.g. quotes, field notes, text excerpts, photographs) to substantiate analytic findings	3-8
**Discussion**			
Intergration with prior work, implications, transferability and contribution(s) to the field	#18	Short summary of main findings; explanation of how findings and conclusions connect to, support, elaborate on, or challenge conclusions of earlier scholarship; discussion of scope of application /	8-10

For peer review only

Knutsen Glette M, *et al. BMJ Open* 2019; 0:e031297. doi: 10.1136/bmjopen-2019-031297

		generalizability; identification of unique contributions(s) to scholarship in a discipline or field	
Limitations	#19	Trustworthiness and limitations of findings	10
**Other**			
Conflicts of interest	#20	Potential sources of influence of perceived influence on study conduct and conclusions; how these were managed	11
Funding	#21	Sources of funding and other support; role of funders in data collection, interpretation and reporting	11

The SRQR checklist is distributed with permission of Wolters Kluwer © 2014 by the Association of American Medical Colleges. This checklist was completed on 26. April 2019 using https://www.goodreports.org/, a tool made by the EQUATOR Network in collaboration with Penelope.ai

For peer review only
